# Estimating Exceptionally Rare Germline and Somatic Mutation Frequencies via Next Generation Sequencing

**DOI:** 10.1371/journal.pone.0158340

**Published:** 2016-06-24

**Authors:** Jordan Eboreime, Soo-Kung Choi, Song-Ro Yoon, Norman Arnheim, Peter Calabrese

**Affiliations:** Molecular and Computational Biology Program, University of Southern California, Los Angeles, CA 90089–2910, United States of America; Queen Mary Hospital, HONG KONG

## Abstract

We used targeted next generation deep-sequencing (Safe Sequencing System) to measure ultra-rare *de novo* mutation frequencies in the human male germline by attaching a unique identifier code to each target DNA molecule. Segments from three different human genes (*FGFR3*, *MECP2* and *PTPN11)* were studied. Regardless of the gene segment, the particular testis donor or the 73 different testis pieces used, the frequencies for any one of the six different mutation types were consistent. Averaging over the C>T/G>A and G>T/C>A mutation types the background mutation frequency was 2.6x10^-5^ per base pair, while for the four other mutation types the average background frequency was lower at 1.5x10^-6^ per base pair. These rates far exceed the well documented human genome average frequency per base pair (~10^−8^) suggesting a non-biological explanation for our data. By computational modeling and a new experimental procedure to distinguish between pre-mutagenic lesion base mismatches and a fully mutated base pair in the original DNA molecule, we argue that most of the base-dependent variation in background frequency is due to a mixture of deamination and oxidation during the first two PCR cycles. Finally, we looked at a previously studied disease mutation in the *PTPN11* gene and could easily distinguish true mutations from the SSS background. We also discuss the limits and possibilities of this and other methods to measure exceptionally rare mutation frequencies, and we present calculations for other scientists seeking to design their own such experiments.

## Introduction

*De novo* mutations in somatic cells and the germline have a major impact on human health. Estimating these rare mutation frequencies using high-throughput DNA sequencing (NGS) is limited by the high technical error rates (non-biological processes) of 10^−2^ to 10^−3^ per nucleotide sequenced. Vogelstein and colleagues developed a strategy, called the Safe-Sequencing System (SSS) with the potential to trump this high error rate [1]. Using SSS, the average mutation frequency per base pair ranges from ~10^−4^–10^−5^, a vast improvement over the normal technical error rates. Accurate estimates of mutation frequencies at rates between 10^−2^ and 10^−4^ could be of great value in some circumstances. Knowing the background of each specific type determines the sensitivity with which *in vivo* mutations of any type can be detected in any DNA sample. We estimated the SSS background for each of the six possible individual mutation types and found some that are closer to 10^−6^–10^−7^.

We performed SSS experiments using DNA from testis tissue on segments from three different human genes (*FGFR3*, *MECP2* and *PTPN11)* using slightly different protocols. The results established the frequencies for each one of the six base-specific mutation types (e.g., C>T/G>A). For comparison, the human germline mutation rate, derived using many different methods, yields an average mutation frequency per base of ~10^−8^ (10^−7^ at CpG sites) [2]. SSS experiments, like almost all high-throughput methods, is subject to background errors [3]. Our experimental data provides a sensitivity limit for mutation detection at the base pair level using SSS, and we explore these sources of error.

We discuss the basis for understanding and evaluating the SSS results, the capabilities of this approach at the present time and SSS’s remaining challenges for enhancing exceptionally rare mutation detection. We also compare SSS to a number of different methods and explain why they all currently suffer from problems that limit average estimates per site to the range of 10^−4^ to 10^−6^ but for reasons that differ among them.

## Results

**Fig 1:**
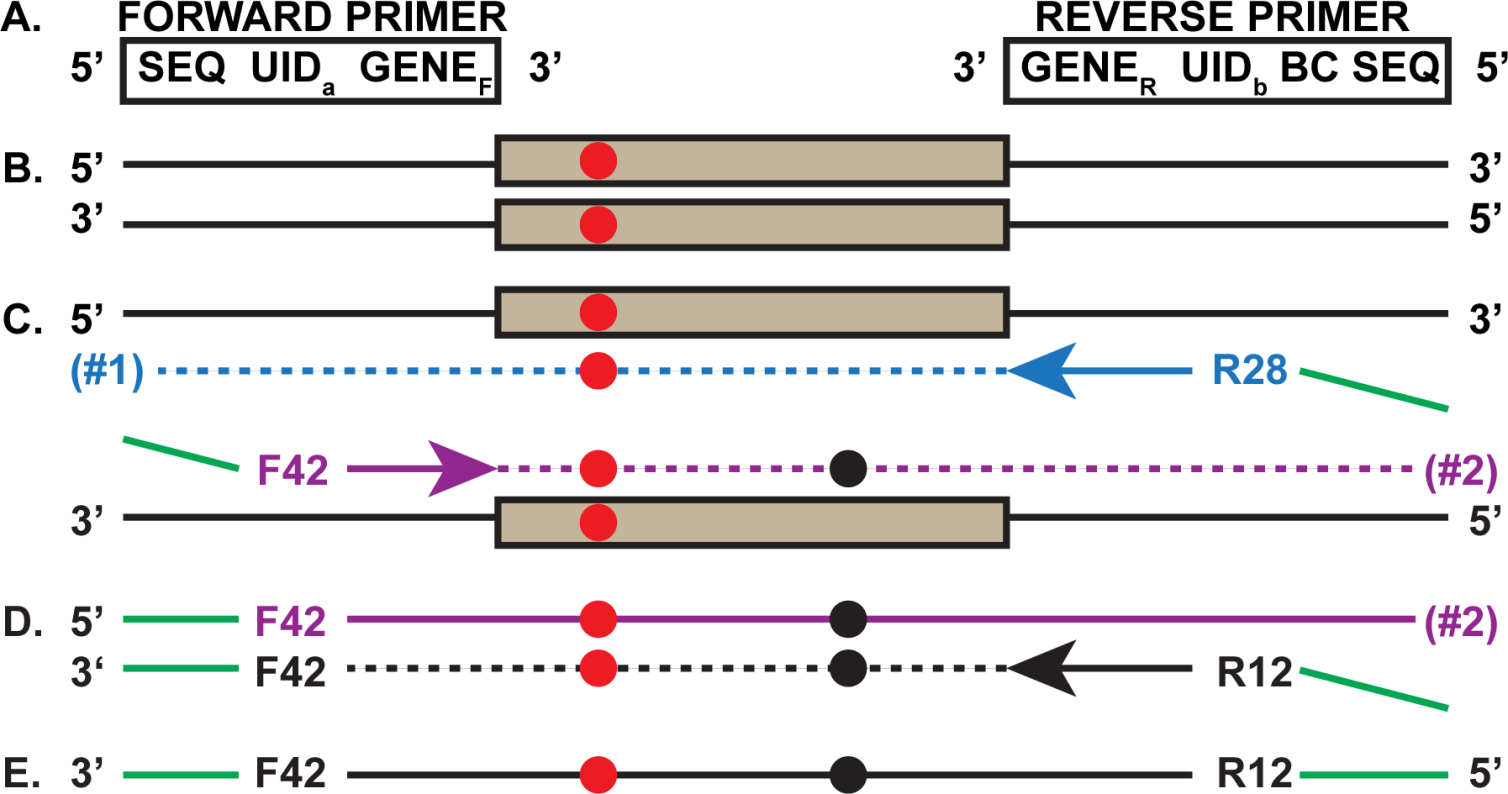
First PCR Round of the SSS strategy. **a.** Two primers (forward and reverse) are used for the first two SSS PCR cycles (all primers are listed in S1 Fig). Each primer (5’ to 3’) contains a Sequencing primer region (SEQ), a Unique Identifier (UID) and the forward (GENE_F_) or reverse (GENE_R_) versions of the gene specific DNA sequences. Only the reverse primer contains a barcode for multiplex analysis (BC). **b.** Duplex genomic DNA fragment carrying a gene target that experienced an *in vivo* germline mutation (red circle). **c.** The two new daughter strands of the first denaturation, primer hybridization and primer extension. The F_42_ and R_28_ represent one of the many UIDs on the forward and reverse primers, respectively. The blue arrow indicates the direction of the reverse primer extension that includes the specific target region (blue dashes) and extends through the target as does the purple forward primer on the other strand (purple dashes). The green overhang at the extreme 5’ ends will anneal to the primers used in the second PCR round. Two different extension products are created (#1 and #2). Notice that extension product #2, with the already existing *in vivo* mutation is (for convenience only) also shown to experience a new *in vitro* mutation (black circle). In the subsequent steps, for simplicity, we only follow one of the two extension products (#2). **d.** The second cycle with the same primer mix, invariably involves a primer (black) with a different UID (R_12_) and copies DNA strand #2 (black dashes). Subsequent single strand-specific exonuclease digestion removes all the initial PCR primers, trims the 3’ DNA overhangs from the second cycle primer extension products and any remaining single-stranded genomic DNA. **e.** Importantly, F_42_-R_12_ is tagged by different UIDs at each end and is then subjected to the second round of PCR (see Fig 2).

**Fig 2:**
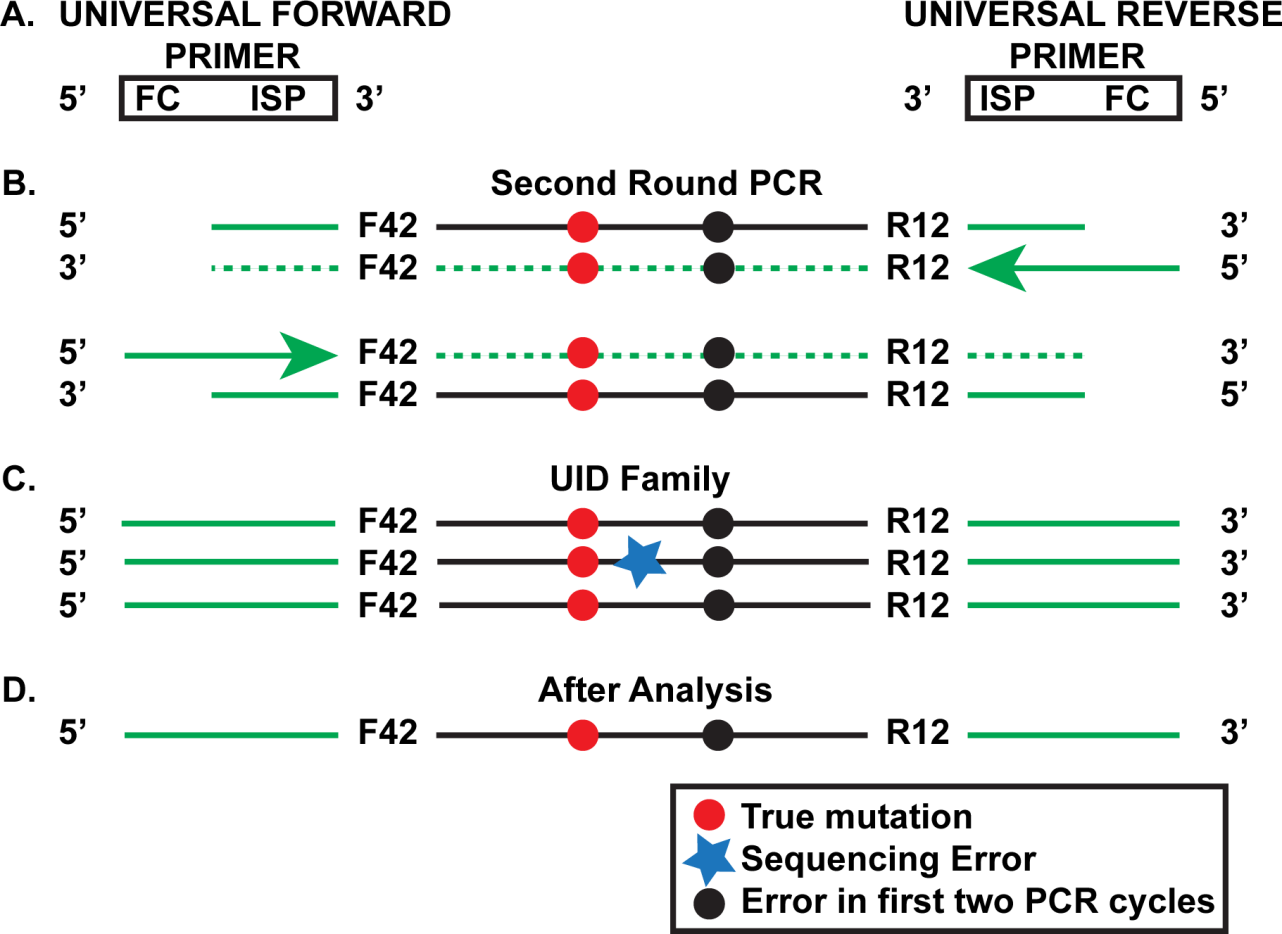
Second Round of the SSS Strategy. **a.** Universal forward and reverse primers are used to amplify the product made in the first round of PCR (see Fig 1E). The primers contain the Illumina Sequencing primer (ISP), which is partially complementary to the Sequencing primer region of the primers used in the first two PCR cycles (Fig 1A). The universal primers also contain the complement of the Flow cell grafting sequence (FC). **b**. Products of the first few cycles of the second round using the template shown in Fig 1E. **c**. Further amplification for 28–30 additional cycles leads to the creation of UID families of which the one shown is representative. The original genomic mutation and the mutation that arose during the first two SSS PCR cycles are both present in this particular family. Notice that one of the family members has accumulated an additional *in vitro* mutation (black star) due to events during the second PCR round or the final sequencing step. **d.** Analysis of the UID family is able to eliminate the latter error from consideration.

When targeting specific DNA segments using SSS, a first round of two initial PCR cycles (Fig 1) uniquely tag each starting DNA molecule (one PCR cycle is defined as a denaturation step, followed by primer annealing and finishing with polymerase extension). The primers contain a target-specific sequence, a string of randomized nucleotides called the Unique IDentifier (UID), and a short universal sequence required for Illumina sequencing. The two cycles are followed by a second round of PCR that specifically expands the target population using primers complementary to the short universal sequence to form the SSS library. Using a large family of different UID sequences (~10^12^) in the first two cycles allows each original target genomic DNA molecule to be distinguished from the other starting target molecules. Importantly, PCR descendants of any one original genomic target molecule will share the same UID sequence (Fig 2). If only a small fraction of the final sequencing reads with the same UID has a mutation then it is most likely due to DNA isolation, library preparation or some other NGS-related error, while a high proportion with the same mutation likely means the mutation was present in the original genomic DNA molecule [1].

We performed the SSS using paired-end sequencing. For the *PTPN11* and *MECP2* experiments the SSS primers were chosen close enough together such that paired reads #1 and #2 overlapped for 32 and 23 base pairs, respectively. This overlap provides an opportunity to measure aspects of the factors that contribute to the overall SSS error [4]. Since the paired reads #1 and #2 are sequencing (in opposite directions) a single DNA strand derived from the final sequencing library, any disagreement between these reads is due to a mistake during the ultimate sequencing step and not an error during PCR amplification while preparing the library. Consider a read pair where both strands each have a high quality score. If, at a specific nucleotide site, the expected complementary base pair is not detected, and one of these reads agrees with the reference sequence, then we assume the other read is in error and arose during sequencing. Three testis pieces were used to derive nine *PTPN11* libraries from a 36 year old donor (#62923). Thirty-two testis pieces from a 68 year old donor (#60891) were the source of 32 *MECP2* libraries. Table 1 shows how the average sequencing error per base pair depends inversely on the Illumina-defined read quality scores. For all of the values in the table, the upper limit of the 95% confidence interval is less than a factor of two greater than the value. For the rest of the paper we only consider those reads where the bases in the barcode and UID have quality scores 32 or greater.

**Table 1:** Sequencing error as a function of quality score.

Quality score	Sequence error
1	1.9 x 10^−3^
5	6.1 x 10^−4^
14	1.3 x 10^−4^
21	5.9 x 10^−5^
26	2.3 x 10^−5^
32	5.3 x 10^−6^
36	1.0 x 10^−6^
39	3.9 x 10^−7^

Sequencing errors determined by the overlap between reads #1 and #2 for the *PTPN11* (testis #62923) and *MECP2* (testis #60891) experiments combined (41 libraries). Sequencing error probabilities are for base pairs with quality scores greater than or equal to the value in the first column.

### UID analysis

Reads that have the same UID are assumed to have descended from the same strand of the original genomic DNA molecule. We define a UID family as a collection of at least 3 reads (see Methods) with the same UID. A super-mutant is a base pair where 95% [1] or more of the reads in a UID family with quality scores 32 or greater (at that base pair) agree with each other and disagree with the reference sequence. The idea is that super-mutants are mutations that were present in the original genomic DNA, however these super-mutants can also be erroneously created during library construction and may be indistinguishable from a real mutation (discussed below). The mutation frequency at a site is the number of UID families with a super-mutant at that site divided by the total number of UID families.

From Table 1, if the quality score is 32 or greater, the probability of a sequencing error per site is 5.3x10^-6^. The probability that 3 of 3 reads have a sequencing error at the same site is (5.3x10^-6^)^3^ = 1.5x10^-16^. To erroneously create a super-mutant all the sequencing errors have to incorrectly call the same base, so assuming that each of the three misincorporated base types are equally likely, the probability that 3 of 3 reads have the same sequencing error at the same base pair is (5.3x10^-6^)^3^x(1/3)^2^ = 1.7x10^-17^. This probability is less than the probability of other possible mistakes. For example, the Phusion DNA polymerase has an error rate of 4.4x10^-7^ per site per cycle (New England Biolabs, unpublished data and [1]). The probability of a super-mutant in UID families with more than three members due to sequencing errors is even smaller. Therefore a super-mutant is unlikely due to sequencing errors.

**Fig 3:**
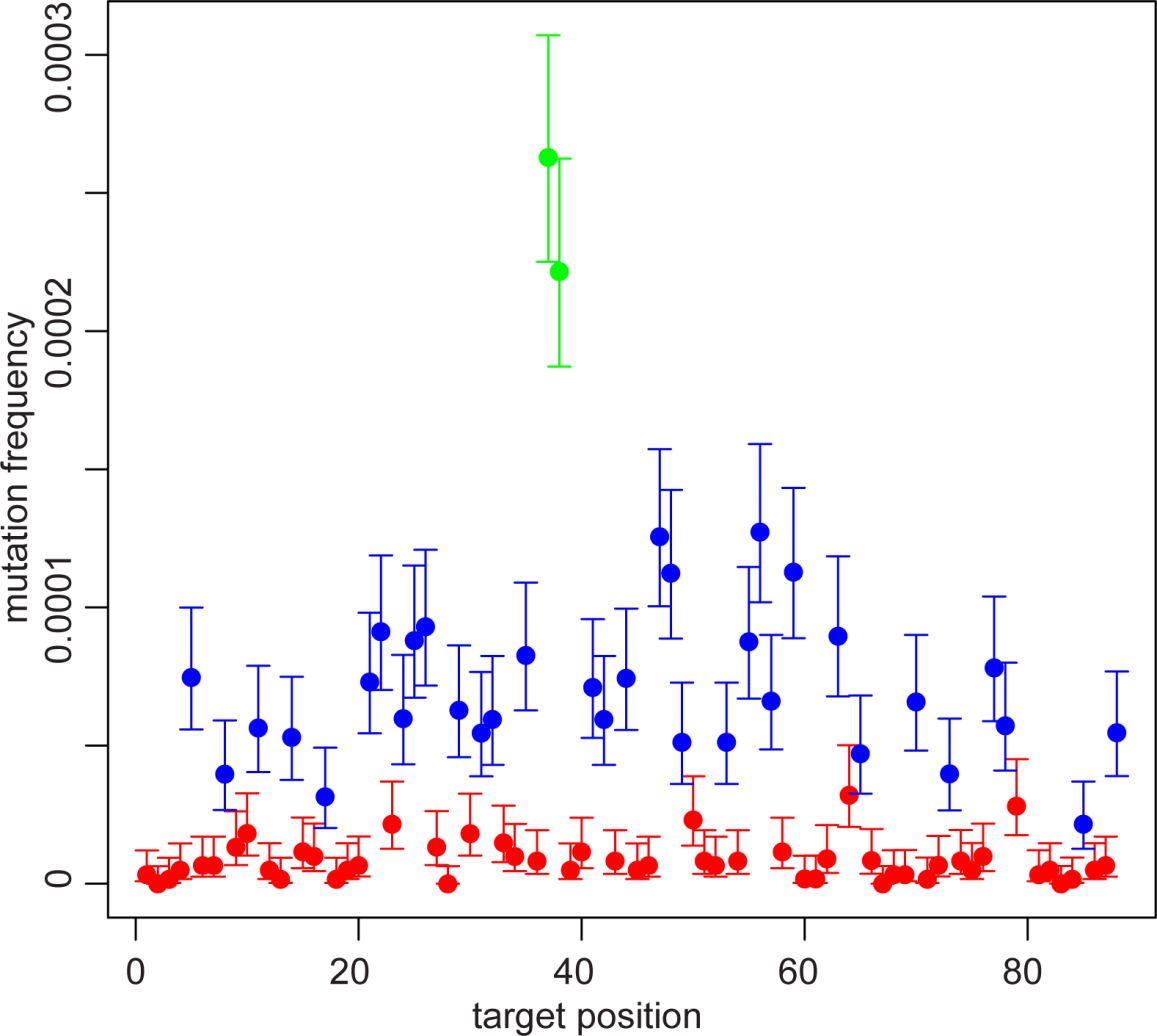
*PTPN11* mutation frequencies color-coded by reference base. Each dot represents the average of 9 different libraries from three testis pieces. Red indicates an A or T base, blue indicates a C or G (non-CpG) base, and green indicates a CpG. The mutation frequency is the sum of all mutations at that site, so, e.g., if a site is a C, the mutation frequency is the sum of the C>A, C>G, and C>T frequencies at that site. The 95% confidence interval for each position is also shown. The data on one base pair (at position 80; c.922) has not been included since it has a much greater mutation frequency; for an explanation, see below.

We carried out UID analysis on the *PTPN11* data. We only considered those base pairs that were not part of the primers, since the estimates at primer sequences would also include the errors introduced during primer synthesis [1]. The average mutation frequency (over all 93 bp and the 3 testis pieces, nine libraries total) at these base pairs was surprisingly high at 3.7x10^-5^ per site. However, the mutation frequency strongly depended on the identity of the base. For a base pair where the reference was an A or T the average mutation frequency was 8.7x10^-6^, while for base pairs where the reference was a C or G the average was 8.0x10^-5^. We observed a similar difference based on the identity of the base when we tested this assay on blood DNA, indicating that this difference is not an artifact of studying germline cells (S1 Table). When we further analyzed data obtained from somatic DNA [1] (kindly provided by Isaac Kinde), we similarly observed a difference (3-fold) in the region of the *CTNNB1* gene these researchers studied; this region did not contain any CpG sites, perhaps contributing to the smaller difference. Fig 3 demonstrates this discrepancy for *PTPN11*. Further investigation revealed that the C>T/G>A mutations and G>T/C>A mutations were responsible for this higher average.

This pattern was repeated for both the *MECP2* (44 base pairs) and *FGFR3* (73 base pairs) experiments (S2 and S3 Figs, respectively). The 64 *FGFR3* and *MECP2* libraries were each made from DNA isolated from 32 different pieces of donor #60891’s testis. Combining the data on all three genes (73 total libraries), Table 2 shows that the C>T/G>A and G>T/C>A mutations are about a factor of 10 more common than the other types of mutations. For all of the values in the table, the width of the 95% confidence interval is less than 50% of the value. The absolute value of the background mutation frequencies in the *FGFR3* experiment were slightly less than for the other two experiments but the relative levels for each base type were the same (S2 Table shows the different mutation type frequencies for the three experiments separately). Except for the *PTPN11* c.922 base pair (a known high frequency disease site), all the other base pairs in the three experiments showed little variation in the mutation frequencies among the different testis pieces.

**Table 2:** Mutation frequency as a function of mutation type.

Mutation type	Mutation frequency
A>C/T>G	4.4 x 10^−7^
C>G/G>C	1.1 x 10^−6^
A>T/T>A	1.4 x 10^−6^
A>G/T>C	3.3 x 10^−6^
C>T/G>A (non-CpG)	1.7 x 10^−5^
G>T /C>A	2.2 x 10^−5^
C>T/G>A (CpG)	7.4 x 10^−5^

Data for the *PTPN11*, *MECP2*, and *FGFR3* experiments combined. Unlike Fig 3, the mutation frequencies are not the sums of all three possible mutations at each base pair.

### Detecting true germline mutations above the SSS background

We previously measured the germline frequencies of the *PTPN11* c.922A>G Noonan syndrome disease mutation in 192 testis pieces (32 pieces in each of 6 slices comprising an entire testis) by targeting this nucleotide with PAP, a version of allele-specific PCR [5, 6]. Among these we selected three adjacent testis pieces for SSS that varied in the c.922A>G mutation frequency. With PAP, the first testis piece (testis #62923, slice 5, piece #18, see [7] for details) had 0 mutations per target site in 5,977,500 studied genomes. As measured by SSS, this piece had 0 super-mutants at that site in 49,655 UID families. The second (piece #19) had a c.922A>G mutation frequency of 3.7x10^-4^ when measured by PAP (close to the average frequency of all pieces for this particular testis [7]), and 1.1x10^-4^ when measured by SSS. The third piece (#20) had the highest disease mutation frequency for this testis. When measured by PAP the mutation frequency was 2.2x10^-2^ and using SSS it was 7.3x10^-3^. Across this wide range of c.922A>G mutation frequencies, the estimates for PAP and SSS were very similar. Our estimate of the A>G/T>C background (Table 2), averaged over all three gene segments, was 3.3 x 10^−6^. These results suggest that A>G/T>C genomic mutations could be analyzed accurately if their occurrence were > 6.6 x 10^−6^ given the large sample size of UID families.

### Test for DNA damage prior or during SSS

The C>T/G>A and G>T/C>A SSS background mutation frequencies may be elevated over their respective genome average mutation rates due to prior DNA damage [8]. Specifically, cytosine>uracil (C>U) or 5’methylcytosine>thymine (C>T) deamination or guanine>8-oxoguanine (G>8-oxoG) oxidation can lead to U:G, T:G or 8-oxoG:C base-mismatches in the double stranded template DNA, respectively. Any pre-existing mismatch can be converted to a full mutation during the first two SSS PCR cycles and produce what appears to be a real mutation. To distinguish between whether the DNA samples we used for SSS contain enough pre-existing mismatches to account for the high observed SSS background frequency of C>T/G>A or G>T/C>A mutations, or whether SSS itself was causative, we proposed a variant to the SSS method we call the “Separate method.” This new method separately estimates the mutation frequencies on the coding and non-coding strands of the initial template.

**Fig 4:**
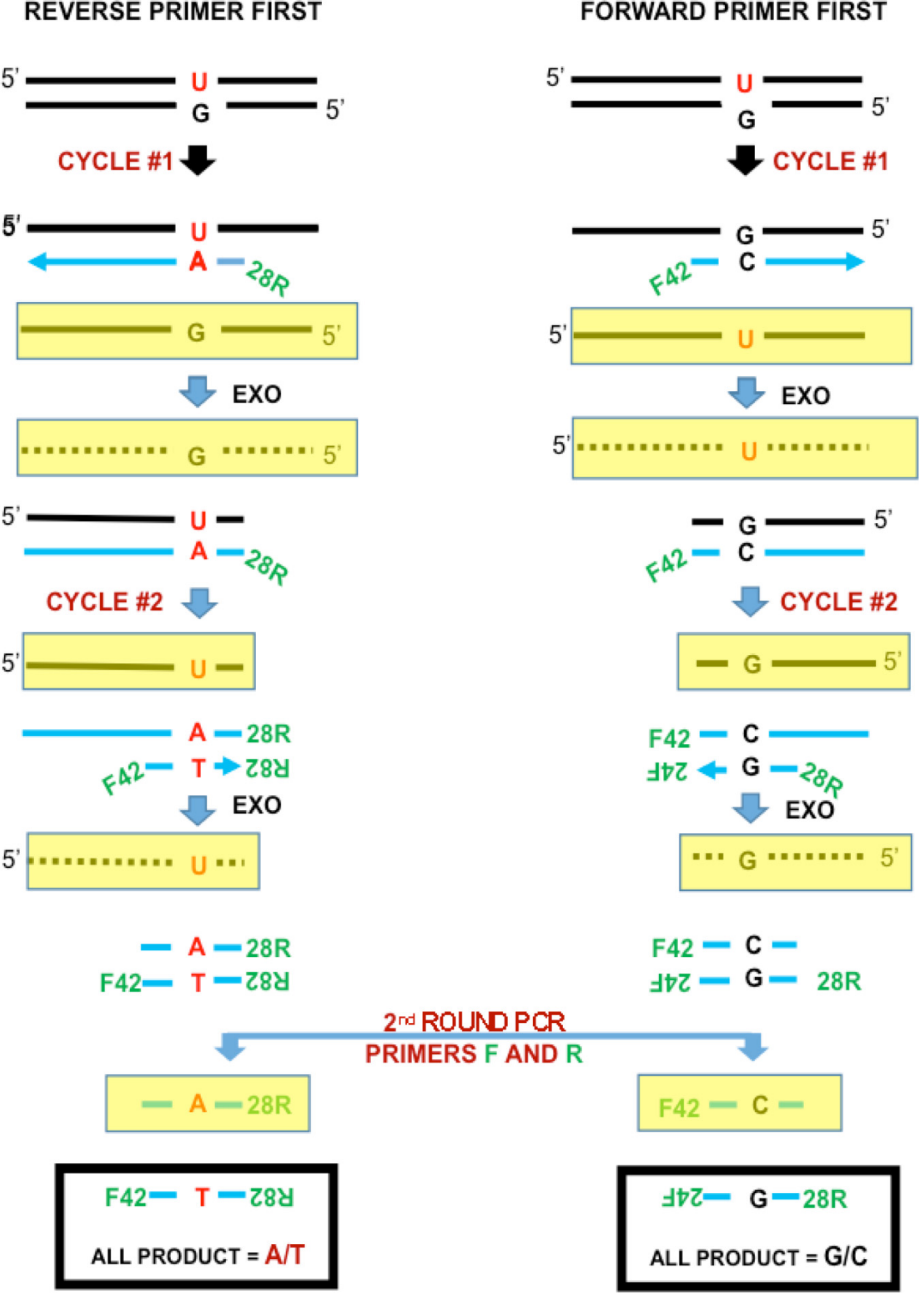
Separate method and pre-existing mismatches. The Separate method separately amplifies the coding and non-coding strands. The aliquot on the left-hand side only amplifies the top strand, while the aliquot on the right-hand side only amplifies the bottom strand. The yellow rectangles indicate strands that are not primer extension products and are removed by exonucleases. For the original molecule shown with a pre-existing U:G mismatch, the Separate method detects a C>T mutation on the left-hand side and no mutation on the right-hand side.

Fig 4 illustrates the Separate method (also see S4 Fig). We took two aliquots from each of the three *PTPN11* DNA samples studied above. The first of the aliquots from each piece underwent one PCR cycle with only the forward SSS *PTPN11* primer present to extend only one of the two target *PTPN11* DNA strands (coding or non-coding). A single strand specific 3’ to 5’ exonuclease (Exo1) was then added to destroy any single-stranded DNA leaving only the portion of the *PTPN*11 target strand made double-stranded by the first primer extension step. Exo 1 also removed any remaining forward primer, the original *PTPN11* target strand not complementary to the forward primer and the bulk of any remaining non-target genomic DNA. The remaining product then underwent a second PCR cycle with the reverse *PTPN11* primer present; exonuclease was again added to destroy the single-stranded DNA. The procedure for the first two PCR cycles used on the second aliquots were the same except the reverse *PTPN11* primer was used first and the forward *PTPN11* primer second. The two subsamples from the same testis piece were mixed (each was identified by a different bar code) followed by the usual second round SSS library amplification procedure. Ideally, the first aliquot amplified only one of the original two DNA strands in the first two cycles and the second aliquot amplified only the other DNA strand.

Fig 4 also depicts what happens to an already existing pre-mutagenic U:G mismatch at a C:G site. The left-hand side of Fig 4 shows the aliquot in which only the top strand participates: the molecule shown has a U at this site on the participating strand, but most other molecules in this aliquot would have a C. After the first two PCR cycles the molecules descended from this particular molecule will have a T:A at this site, where the T is on the strand which had harbored the U. Since only the top strand was involved, the Separate method will classify this mismatch as a C>T mutation. (In contrast, the SSS by itself would be unable to distinguish between a C>T and a G>A mutation). The right-hand side of Fig 4 shows the aliquot in which only the bottom strand participates. For the molecule shown, the Separate method will not detect a mutation at this site (a T:A base pair, not a mismatch, at this site in the original duplex would have been detected in the right hand aliquot as a G>A mutation).

By combining all of the C:G sites (regardless of whether they are on the coding or non-coding strand), we can separately estimate the average C>T or G>A mutation frequencies. If a substantial fraction of the DNA templates are carrying pre-existing U:G or T:G base mismatches, then the overall C>T mutation frequency will be substantially greater than the G>A frequency. Similarly, significant amounts of 8-oxoG:C mismatches on the original molecules will result in the G>T mutation frequency being substantially greater than the C>A frequency. Those mutations that are not enhanced by oxidation or deamination (e.g., A>C and T>G) would not be expected to show a sizable difference.

Table 3 shows the mutation frequencies for each mutation type estimated by the Separate method. This data mirrors the observation from Table 2 that there is a stark difference between C>T as well as G>T mutations (lasts three rows) and the other mutation types (first four rows). Part of the novelty of the Separate method is that it independently estimates mutation frequencies that are indistinguishable by the SSS method: these pairs of previously indistinguishable mutations are listed on the same rows of Table 3. If there were a substantial amount of pre-existing mismatches (e.g., due to deamination or oxidation) then the mutation frequency of one mutation would be significantly greater than the other mutation on the same row. For all of the rows in Table 3, the difference in mutation frequencies between those mutations on the same row is less than a factor of two. To take one example, the A>G mutation frequency of 6.7x10^-6^ is close to the T>C frequency of 6.5x10^-6^. The p-value of 0.47 indicates that there is no statistically significant difference between these two frequencies, implying that there is not a substantial amount of pre-existing mismatches that would increase the frequency of one mutation over the other. As expected, all of the other mutations that are far less likely to be enhanced by deamination or oxidation (in rows 1–4) also lack any hint of statistical significance.

**Table 3:** Mutation frequencies estimated by the Separate method.

Mutation type	Mutation frequency	Mutation type	Mutation frequency	p-value
A>C	8.3x10^-7^	T>G	1.3x10^-6^	0.80
C>G	3.1x10^-6^	G>C	2.5x10^-6^	0.30
A>T	2.3x10^-6^	T>A	3.5x10^-6^	0.87
A>G	6.7x10^-6^	T>C	6.5x10^-6^	0.47
C>T (non-CpG)	3.7x10^-5^	G>A (non-CpG)	2.8x10^-5^	0.01
G>T	4.7x10^-5^	C>A	3.7x10^-5^	0.02
C>T (CpG)	3.3x10^-4^	G>A (CpG)	1.6x10^-4^	0.0001

Data from the *PTPN11* experiment.

For those mutations that are more likely to be enhanced by deamination or oxidation (last three rows), the difference varies from being statistically significant to marginally significant. At C:G base pairs (not in a CpG site), the C>T mutation frequency of 3.7x10^-5^ is slightly greater than the G>A frequency of 2.8x10^-5^. The p-value of 0.01 is marginally significant (to be significant at the 0.05 level after the Bonferroni correction for seven tests requires a p-value less than 0.007). Similarly, the G>T mutation frequency is slightly greater than the C>A frequency at marginal statistical significance. At the one CpG site present in the region of *PTPN11* studied, the C>T mutation frequency of 3.3x10^-4^ is greater than the G>A frequency of 1.6x10^-4^, with a statistically significant p-value 0.0001. However, the small intra-row differences are not sufficient to cause the large difference in mutation frequencies between C>T and G>T mutations and the mutations in the first four rows of Table 3. For example, in a CpG the C>T mutation frequency is only two times greater than the G>A frequency. But both of these mutation frequencies are as much as two orders of magnitude greater than the frequencies in the first four rows. Further, the G>A mutation is not even expected to be enhanced over C>T by pre-existing mismatches.

The most likely explanation remaining for the high background frequency of those mutations in the last three rows of Table 3 is the SSS method itself. Mutations that arise during the first two cycles of PCR cannot be corrected using the SSS UID approach (and only mutations that arise after the first two PCR cycles can be corrected). S5 and S6 Figs show the mutational effects of DNA damage that occur during these first two cycles, most likely due to heat-generated deamination and oxidation. Assuming DNA damage is equally likely to arise during these two cycles, such damage will affect the two separate aliquots equally. The increase in the C>T mutation frequency will then be balanced by the increase in the G>A frequency (the same logic applies to the G>T and C>A frequencies).

Since DNA damage during the first two PCR cycles affects the pairs of mutations on the same row of Table 3 equally, the relative differences between these mutation frequencies indicates the fractions of mutations due to pre-existing mismatches. For the C>T (non-CpG), G>T, and C>T (CpG) mutations these fractions are 0.26, 0.28, and 0.53, respectively. Using the bootstrap, the 95% confidence intervals for these fractions are (0.03–0.43), (0.02–0.60), and (0.30–0.70). Thus, eliminating all pre-existing mismatches would not reduce the mutation frequencies for these mutation types to the levels observed for the mutation types in the first four rows of Table 3.

Supporting this conclusion, experiments in yeast using a novel high-throughput sequencing method [9] found that treating yeast DNA with DNA repair enzymes before initiating sequencing library construction did not reduce the observed excess of C>T/G>A and G>T/C>A mutation types. However, when the yeast sequencing library amplification step was carried out in the presence of DNA repair enzymes, the level of these errors was considerably lowered (a more detailed analysis is found in the Discussion).

### PCR errors

If a wrong base is incorporated by the DNA polymerase during primer extension in either of the first two PCR cycles, when the UIDs are being attached, then this incorrect base will be shared by all members of a single UID family to produce an erroneous super-mutant. Mistakes during the second round PCR step can be detected. The Phusion polymerase has an error rate of 4.4x10^-7^ per cycle per base pair, so the background mutation rate estimated for SSS is at least 8.8x10^-7^. Tables 2 and 3 show a greater mutation frequency than this minimal background, especially for the C>T/G>A and G>T/C>A mutation types.

We propose a method to independently estimate the PCR error rates for each of the different mutation types. This method estimates the new errors that arise beginning in the second round of PCR (after the first two PCR cycles are completed); we assume then that these error rates will be the same as was in the first two PCR cycles where false super-mutants (that cannot be distinguished from a true genomic mutation) are created. In order to minimize sequencing errors, we only consider those bases in the overlap region of the *PTPN11* and *MECP2* experiments for which reads #1 and #2 agree with each other (and the quality scores are 32 or greater). The probability that there is a sequencing error in both reads #1 and #2 at the same base pair is (5.3x10^-6^)^2^ = 2.8x10^-11^, which is substantially less than the PCR error rate. We measure how often one member of a UID family has a mutation that is not present in any of the other family members. These mutations are not super-mutants. The most likely explanation is that such a PCR error occurred after the first two cycles. Due to the initial exponential increase in the number of molecules during PCR amplification, the lineage of a UID family forms a “star phylogeny” [10] where all the members of the UID family diverge near the beginning of the second round of PCR. In order for a PCR error to be present in only one member of a UID family, this error must have occurred in one of the long branches after this divergence that is part of the lineage of only one member. Thus, for a UID family observed with two members, there were approximately 60 of the second-round cycles (~30 cycles on each branch) where a PCR error will create a mutation found in only one member. Likewise for a UID family with n members, there are approximately 30xn cycles (~30 cycles on each branch) where a PCR error will create a mutation in only one of the n members. In order to estimate the per cycle PCR error rate separately for each mutation type we then fit a linear model where the independent variable is the UID family sizes ranging from 2 to 10 and the dependent variable is the frequency that there is a mutation in only one member of a UID family of this size. For all six mutation types, we obtained an excellent fit for a linear model.

Table 4 shows the PCR error rate estimates (the standard error for all these estimates is less than 17% of the estimates). The PCR error rate estimates vary based on mutation type, however, this variation is not as extreme as the variation in mutation frequencies. For example, in Table 2 the ratio of the average mutation frequencies for the C>T/G>A and G>T/C>A mutations to the average mutation frequencies for the four other mutation types is 17, while in Table 4 the ratio of the average PCR error rates for the C>T/G>A and G>T/C>A mutations to the average PCR error rates for the four other mutation types is only around 2. In Table 4, we also calculate the ratio of mutation frequencies (from Table 2) to PCR error estimates. We expect this ratio to be around two since a PCR error in the first two cycles will create a false super-mutant. For the A>C/T>G, C>G/G>C, A>T/T>A, and A>G/T>C mutation types this ratio ranges from 1.9 to 6.4, but for the C>T/G>A and G>T/C>A mutation types this ratio ranges from 16.9 to 28.5. The larger ratios suggests (contrary to our original assumption) that there is a higher PCR error rate during the first two PCR cycles for the C>T/G>A and G>T/C>A mutation types.

**Table 4:** Estimates of the PCR error rate for the different mutation types.

Mutation type	PCR error rate (per site per cycle)	Mutation frequency / PCR error rate
A>C/T>G	6.9 x 10^−8^	6.4
C>G/G>C	3.0 x 10^−7^	3.7
A>T/T>A	7.5 x 10^−7^	1.9
A>G/T>C	1.5 x 10^−6^	2.2
C>T/G>A (non-CpG)	1.0 x 10^−6^	17.0
G>T/C>A	1.3 x 10^−6^	16.9
C>T/G>A (CpG)	2.6 x 10^−6^	28.5

### Utility ratios

Table 5 shows the statistics for the number of initial molecules, the total number of reads, the number of quality reads (reads such that all base pairs in the barcode and UID have quality scores greater than 32), and the number of UID families for the three experiments. Quality reads are wasted both in unnecessarily large individual UID families and if there are fewer than three reads with the same UID (these reads by our criteria do not form a UID family). UID family sizes are larger for the *PTPN11* experiment than for the *MECP2* and *FGFR3* experiments. For the *PTPN11* experiment the average UID family size is 105 and 18% of families have 100 or more members, while for the *MECP2* and *FGFR3* experiments the average family size is 7.1 and 8.1, respectively, and for both of these experiments less than 0.1% of families have 100 or more members. Strikingly, for the *PTPN11* experiment, the largest family has over 50,000 members while for the *MECP2* experiment the largest family has 287 members. On the other hand, for the *PTPN11* experiment only 1% of reads are wasted for having less than three reads with the same UID, while for the *MECP2* and *FGFR3* experiments 32% and 30%, respectively, of reads are wasted for this reason. To understand this variation we introduce the utility ratio.

**Table 5:** Read and UID family statistics for the three experiments.

	*PTPN11* experiment	*MECP2* experiment	*FGFR3* experiment
Initial molecules	2.3 x 10^6^	3.2 x 10^7^	3.2 x 10^7^
Pairs aligned reads	8.3 x 10^7^	1.3 x 10^7^	4.6 x 10^7^
Quality reads^a^	6.4 x 10^7^	7.6 x 10^6^	2.8 x 10^7^
UID families	6.1 x 10^5^	7.3 x 10^5^	2.5 x 10^6^
Utility ratio^b^	106	10.4	11.4
Average family size	105	7.1	8.1
Fraction families with sizes 3–10	36%	85%	80%
11–99	46%	15%	20%
100 +	18%	< 0.1%	< 0.1%
Fraction reads with less than 3 reads with same UID	1%	32%	30%

^a^ Quality reads: number of paired aligned reads such that all base pairs in the barcode and UID have quality scores greater than 32.

^b^ Utility ratio: Quality reads / UID families

We define the utility ratio as the ratio of quality reads to the number of UID families. The *PTPN11* experiment, which has a utility ratio of 106, then, makes less efficient use of the reads than the *MECP2* and *FGFR3* experiments, with utility ratios of 10.4 and 11.4, respectively. The reason for this difference is that the ratio of quality reads to initial molecules is 28 for the *PTPN11* experiment, and less than 1 for the *MECP2* and *FGFR3* experiments. A higher fraction of the initial molecules are then represented as UID families for the *PTPN11* experiment than for the other two experiments. The number of UID families cannot be greater than two times the number of initial molecules (during the two cycles of the first PCR round, each initial molecule is assigned two distinct UIDs). When the number of UID families is much less than the number of initial molecules, additional reads will generally contribute to new UID families. As the number of UID families approaches the number of initial molecules, additional reads will generally not yield new UID families but rather increase the size of existing UID family sizes. If all of the UID families have exactly three reads this will be the most efficient use of reads and the utility ratio will be 3. In calculations #7 and #8 in S1 Text we show that the optimal obtainable utility ratio is 5.15. Moreover, this optimum is achieved when the ratio of the number of quality reads to the number of initial molecules is 3.3 (the actual denominator is initial molecules with a UID and barcode attached during the first two rounds of PCR, which may be less than the number of initial molecules, see calculations #7 and #8 in S1 Text). These results depend on using 3 as the minimum family size, but the derivation presented in calculations #7 and #8 in S1 Text enables computations for other minimum family sizes.

### How a UID family with a very large numbers of reads can create spurious super-mutants

**Fig 5:**
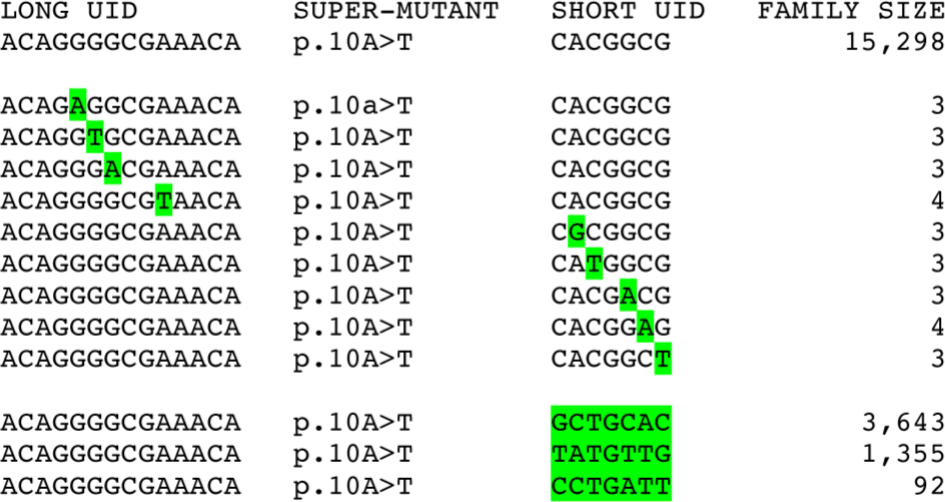
Spurious super-mutants due to very large UID families. One very large UID family on the top row is erroneously counted as twelve additional families. The next nine rows show families with UID sequences that differ at one site from the family in the top row, most likely due to a mistake during PCR amplification. The bottom three rows show families with the same long UID sequence but a different short UID sequence from the family in the top row, most likely due to PCR jumping. All of these families contain the same A>T/T>A super-mutant at the read position 10 bases from the end of the UID (p.10A>T) erroneously increasing its frequency.

There are several ways that spurious super-mutants can be created during the second round of PCR. These ways are most likely to be associated with the creation of a very large UID family. Fig 5 illustrates examples from the *PTPN11* data. All of the UID families depicted in the figure have an A>T/T>A super-mutant at position 10. The first UID family listed is very large, containing 15,298 reads. The next nine UID families are much smaller, containing 3 or 4 reads, and each of their UID sequences differ from the UID sequence of the first family at exactly one nucleotide position. These nine UID families are most likely created by mistakes in PCR amplification of the UID sequence of the first family (see calculations #2 and #3 in S1 Text), and should not be counted as distinct families.

The next three UID families have the same long UID sequence (from the forward primer) as in the first family, but a different short UID sequence (from the reverse primer) compared to the first family. These three UID families are most likely caused by PCR jumping (see calculation #4 in S1 Text). PCR jumping is when primer extension is incomplete in one cycle and the incomplete PCR product then acts as a primer in a subsequent cycle. Since the UID is split between the two ends of the paired read, such a jumping event will combine the long UID from one family (in this case the first family) with the short UID from a different family to create a new combined UID sequence family. Depending on where the jumping event takes place, a mutation may also be copied into these new UID families. These families should also not be counted as distinct families.

Errors during the second round of PCR caused the first large family to be incorrectly represented by 12 other UID families. This first family has an A>T/T>A super-mutant at nucleotide position 10 which is also present in these 12 other families, thereby erroneously increasing the estimate of the mutation frequency at this nucleotide position. We were able to correct these mistakes by: (1) if multiple families have UID sequences that differ at exactly one nucleotide position we only consider the largest such family, and (2) if multiple families have the same long UID sequence but different short UID sequences we only consider the largest such family. Only a small number of corrections were required, and these all involved very large UID families. In S1 Text, calculations #5 and #6, we show how two mistakes during the second round of PCR amplification can lead not only to over-counting super-mutants but to creating super-mutants that do not correspond to mutations present in any original molecules. We can correct for these mistakes as above, and also found there were few corrections required.

## Discussion

We provide a detailed analysis of the SSS method as applied to germline mutation frequency estimation at three different human genomic regions. Many studies where the whole genome sequences of parents and their children were compared have confirmed previous estimates of the average germline mutation frequency in humans as ~10^−8^ per nucleotide site per generation (reviewed in [2]). These trio NGS experiments do not depend on low background error rates because they compare the sequence of parents with their offspring to detect germline mutations and in each individual very small coverage of each site is enough to establish the validity of the nucleotide at that site (consensus or mutant).

Average mutation frequencies (not base specific) measured by SSS [1] in a fragment from somatic tissue (~10^−5^) are ~20 fold lower than the same authors measured using a standard deep-amplicon sequencing NGS analysis (~2x10^-4^); SSS thus eliminates a significant fraction of mutations induced during normal library preparation and NGS sequencing. However, this SSS study on normal non-germline tissues gave overall mutation rates that were still orders of magnitude greater than the average germline mutation rate, possibly due to the errors reported here.

We used SSS to examine germline cells to estimate the average background mutation rate in order to understand better the types of errors that still remained in the SSS procedure and the potential for overcoming these errors. We compared the results of the SSS method with the mutation frequencies found in our previous study of a Noonan syndrome mutation (c.922G>A) using the highly sensitive allele-specific PAP assay [7]. SSS on three testis pieces gave an excellent correspondence between the two methods. In addition, unlike our previous PAP assay, the SSS method allowed us to examine a large number of base pairs simultaneously. With the exception of the Noonan disease site the average *PTPN11* background mutation frequency over all base pairs was consistent with what had been reported previously for SSS [1]. However, we also examined the mutation frequencies as distributed among the six types of possible mutations (C/T>G/A, etc.) and found a marked difference among them as shown earlier in non-germline tissues [9, 11, 12]. Our SSS study of two additional genomic regions (*FGFR3* and *MECP2*), on each of a total of 64 individual libraries of different testis pieces, gave the same results. A list of possible factors that might lead to errors in SSS experiments was published in the supplemental data accompanying [1]. Highly detailed analysis of our data provide new quantitative insights into the source of many of these errors.

### SSS experimental design

There are multiple issues to consider when designing an SSS experiment. Theoretically, the lowest mutation frequency we can measure at a site by SSS is one divided by the number of UID families covering that site. Thus the properties of the UID families are critical. In S1 Text, we derive and present formulas for calculating the answers to several computational questions. The first concerns the characteristics of the UID sequence. What is the probability two distinct DNA molecules are assigned the same UID? If there is a mistake in the UID sequence (either during sequencing or PCR amplification) what is the probability this mistaken UID is the same as a UID already in use on another DNA molecule? What is the probability one DNA molecule is incorrectly represented by multiple UID families? We also address some ways to erroneously create super-mutants. What is the probability a mistake in one of the early cycles of the second round of PCR creates an erroneous super-mutant? How likely is it that two mistakes during PCR erroneously create a super-mutant? For our experiments, these probabilities are low enough that they are not a problem but for other SSS applications it may be wise to consider these issues.

We have introduced the utility ratio as the ratio of “quality” reads to the number of UID families. For the *MECP2* and *FGFR3* experiments the utility ratio was near 10. The *PTPN11* experiment made less efficient use of reads and this ratio was near 100. The reason for this difference is that for the *PTPN11* experiment there was a higher ratio of reads to initial molecules compared to the other two experiments. In parts #7 and #8 of S1 Text, we provide a formula relating the number of initial molecules, the number of reads, and the number of UID families, and discuss the optimal utility ratio for designing experiments. Depending on the experimental conditions extremely large families can be created (these were rare in the *PTPN11* experiment and non-existent in the *MECP2* and *FGFR3* experiments) which can then create spurious super-mutants, but as discussed in the Results and S1 Text these can be corrected relatively easily.

While it is not necessary, there are advantages to having the paired-end reads overlap. First, this overlap provides a way to measure the sequencing error. One can also use the Illumina quality scores to estimate the sequencing error. In order to achieve a specified false positive rate for the SSS method due to sequencing error, one can either require a relatively small number of reads with relatively high quality scores to form a UID family, or alternatively one can be more lenient with the quality scores but require more reads. We have tried both calculation strategies with no effect on the mutation frequency estimates. One way to decide between these two strategies is to select the strategy that maximizes the number of UID families (which will depend on the particular experiment), in order to lower the threshold of mutation frequency detection and to minimize the confidence intervals of the mutation frequency estimates. A second advantage of having the paired-end reads overlap, is if the overlap includes any target site(s) of interest, then each paired-read will sequence this base pair twice. Therefore, compared to base pairs not in the overlap region, these overlapped base pairs will likely have more reads above the quality score threshold, potentially leading to a larger number of UID families and greater mutation detection sensitivity.

The biggest problem with the SSS and most other NGS methods is that the estimated C>T/G>A and G>T/C>A background mutation frequencies per site are approximately an order of magnitude greater than the estimates for the other mutation types. Although very unlikely, biased non-deamination/oxidation-related C>T/G>A and G>T/C>A misincorporation errors by the Phusion DNA polymerase cannot be ruled out but we found no data on base-specific mutation frequencies for this enzyme.

The combination of results from the Separate method and our PCR error rate analysis (Tables 3 and 4) favor deamination and oxidation errors during the first two PCR cycles when the UIDs are being attached. It is unclear why these two cycles would have a greater error rate than the cycles in the second round of PCR. The high temperature of PCR is a likely source of deamination (heat also acts to produce 8-oxoG [13]). However, while the first two cycles of SSS library production varied among our three target genes in temperature and its duration (compared to the second round PCR cycles; see Methods) these differences were found to have an insignificant effect on C>T/G>A and G>T/C>A mutation rates. For example, the initial denaturation, at 98°C, before beginning the first cycle of PCR is 1 minute for the *PTPN11* experiment and 6 minutes for the *MECP2* experiment, yet there is no difference in the C>T/G>A or G>T/C>A mutation frequencies for these experiments. Our data suggest that additional experiments need to be carried out before we can pinpoint the time and cause of these mutations. Finally, although it is not relevant to SSS, it has also been proposed that certain procedures during classical NGS library preparation (e.g., Covaris shearing) produce G>T/C>A mutations through oxidation [14].

The SSS targeting method is rapid and simple to carry out. It takes one person approximately two weeks to make ~200 libraries. For detecting C>G/G>C mutations or those at an A or T nucleotide, the false mutation rate is no greater than 3.3x10^-6^ per site and, in the case of A>C/T>G, ten-fold less. Mutations associated with many human diseases fall into these four mutation categories and for some studies SSS might be a wise choice. The ability to calculate the efficiency (utility ratio) of converting DNA into UID families is also an advantage. However, if C>T/G>A or G>T/C>A mutations are the targets (average false positive rate 2.6x10^-5^) the attainable error-free detection sensitivity will be 10–30 times (at CpGs) lower than for the rest of the mutation types.

### Alternative mutation rate detection methods

Sawyer and co-authors [9] proposed a different method (circle sequencing) using rolling circle amplification (RCA) of circularized yeast genomic DNA fragments at 37°C without using UIDs. This method applies to whole genome studies and as yet cannot be targeted to a specific region. It can make much more efficient use of the reads than either SSS or DS since RCA puts multiple copies of each original molecule on one read. Then each read is in effect one UID family (and reads are not wasted in families smaller than useful or larger than needed). They also found highly increased genome wide C>T/G>A and G>T/C>A mutations similar to those seen by others and ourselves. Adding *E*. *coli* uracil-DNA glycosylase and *E*. *coli* formamidopyrimidine-DNA glycosylase to the purified yeast DNA before library preparation did not significantly reduce the C>T/G>A and G>T/C>A error rate indicating that purification itself did not induce any deamination or oxidation errors leading to a mismatch. However, after denaturation and circularization of the yeast DNA strands, they found that the excess C>T/G>A and G>T/C>A mutation frequencies could be reduced to levels similar to those observed for the other four mutation types if the two *E*. *coli* DNA glycosylases were present during the 37°C RCA step. The high C>T/G>A and G>T/C>A mutation rates (without added enzymes) must have arisen from events after DNA purification: during ligation mediated circle formation (60°C for a total of 22 hr) and/or during RCA (37°C) but it is not possible to pinpoint whether only one or both steps contributed. Their data is consistent with our evidence for SSS that the majority of DNA damage leading to high background mutation frequencies occurs during the first two cycles of PCR and we suggest that, in the case of circle sequencing, most likely arises during circle formation that is carried out at 60°C prior to RCA.

Shendure and co-authors [12] proposed a single molecule molecular inversion probe [15] method (smMIP) that uses a linear oligonucleotide whose ends anneal to both sides of each target region leaving a gap of ~100 bp. Similar to SSS the oligo contains a unique randomized tag. After gap filling on genomic DNA and ligation, each individual circular target DNA molecule (with its internal tag) is amplified by PCR forming a “tag-defined read group” which corresponds to a UID family. This method has enormous potential for multiplex targeted analysis of different genomic regions simultaneously. By using overlapping read-pairs, they are able to use a relatively small number of reads in each group. Another group [4] also proposed the idea of using overlapping read-pairs to lower sequencing errors (but without molecular tags). smMIP has a per-base error of 2.6x10^-5^ in clinical samples that is similar to what we measured for the SSS background frequency. Like SSS, the smMIP method yields elevated substitution rates for certain mutation types, presumably due to oxidatively damaged guanine (8-oxoG) and spontaneous deamination of cytosine and 5-methyl-cytosine. Interestingly, they observed markedly higher rates in the formalin-fixed, paraffin-embedded (FFPE) samples than the HapMap and fresh clinical samples, suggesting that the FFPE treatment may increase 8-oxoG formation rates.

Giannoulatou et al. employed a very different strategy to measure rare mutation frequencies at one particular 4 base pair target region imbedded in a small DNA segment [16]. They used a restriction enzyme that cut the wild-type target sequence but not any mutant sequence at this target. They size-selected the uncut mutant sequences, spiked in known amounts of controls from individuals that were heterozygous for various mutations at this locus and prepared a classical Illumina sequencing library with barcodes to identify samples (but without random UIDs to identify individual starting molecules). A more complicated statistical analysis than in the other methods compared the number of mutant reads in the samples to the number of mutant reads from the controls to estimate the mutation frequencies at this locus. Similar to the other methods there was a difference in the background frequency based on the type of mutation: 3x10^-6^ for transversions and 10^−5^ for transitions. Since only a subset of the possible mutation types are present at this target it is not possible to make a direct comparison to SSS, but these average background frequencies are similar to what we observed.

The SSS and the above methods cannot tell which strand a mutation arose on, while the Separate method separately measures the average mutation frequencies on the coding and non-coding strands. Schmitt et al. [11] proposed a Duplex Sequencing (DS) scheme that goes one step further by separately keeping track of the individual coding and non-coding strands for each original DNA duplex. A mutation is inferred in the original molecule only if the descendants of both the original coding and non-coding strands are found to produce a super-mutant at a site. It is very unlikely that the same error will independently occur at the same base pair during the PCR amplification of both original strands. (Unlike DS, in the abovementioned methods the two original strands are separated before being marked and their relationship to one another is therefore lost forever.) In their original paper [11], the DS sequencing method was applied only to a small virus genome and purified mitochondrial DNA and so was able to generate very large numbers of UID family equivalents with relatively little starting DNA and NGS sequencing. Encouragingly, they found little difference in the background mutation frequencies of the different mutation types having been able to eliminate virtually all the false positives due to deamination and oxidation. The ratio of reads to duplex nucleotides (comparable to our utility ratio, see Table 5) ranged from 64 to 295 [11]. In a more recent paper [17], the authors modified the method. After ligation of the duplex markers to fragmented human DNA (followed by PCR) they enriched for the target region by specific probe hybridization (NimbleGen). This modification led to a highly purified 2–3 kb segment from total human DNA. However, the DNA sequencing results reported were limited in sensitivity by the low depth of coverage achieved; the minimum mutation frequency per site was only greater than 1/1,000, a depth of ~1,000 duplexes (UID family equivalents) per site in the target region. They suggest carrying out many more identical reactions could solve this problem. However, increasing the depth of coverage 1,000 times, (to achieve a 10^−6^ sensitivity per site, for example) would have to involve substantial increases in the amount of starting DNA and the associated costs of NimbleGen target enrichment and sequencing. Nevertheless, assuming that enough DNA and other resources are available this is an extremely promising approach to minimizing the effect of false positive mutations.

### Applications

There are many situations where enhanced NGS methods for measuring exceptionally rare mutation frequencies would be useful. For example, the level of germline mosaicism and the specific spatial distribution of spontaneous mutations in human testes could provide insight into the cellular and molecular basis of the paternal age effect (for example see [7, 18]). Similarly, more sensitive detection of *de novo* base substitution mutations in easily accessible somatic sources could enhance detection of genetically mosaic parents with a high risk for having an affected child [19]. Also, somatic mosaicism in peripheral blood has been found in 30% of patients with recognized brain malformations and more sensitive methods are desired to extend these findings to other neurodevelopmental diseases [20]. Extra-sensitive NGS methods would also enhance some aspects of cancer diagnosis using tissue biopsies and, in some circumstances, circulating tumor DNA/cells in plasma [21–24]. Similarly, the nature of genetic heterogeneity in individual tumors could be explored in far greater detail (reviewed in [14]). Analyzing the effect of random mutations on retroviral and influenza virus functions [25] and virus strain variation [26] is also benefitting from rare mutation detection.

## Materials and Methods

### Source of DNA

Testes from a 68 year old donor (#60891) and a 36 year young donor (#62923) were supplied by the National Disease Research Interchange (www.ndriresource.org) and we acknowledge the use of tissues procured, by them, with support from NIH grant 2 U42 OD011158. NDRI consent forms and protocols are reviewed and approved annually by the Institutional Review Board at the University of Pennsylvania. NDRI obtains tissue from over two hundred tissue acquisition sites. NDRI rigorously reviews and qualifies all potential procurement sites prior to admitting them into the NDRI network. NDRI requires all sites in their network to:

1) Obtain informed consent in writing from any donor of human tissue (or the next-of-kin thereof) for the use of that tissue for research. 2) Keep a signed consent form on file at the tissue acquisition site. Information is never released to any third party. NDRI audits recovery sites to ensure they are obtaining consent in compliance with all regulations governing that process. 3) Have language in the consent forms that indicates that the tissue is an anatomical gift for which no compensation is given. NDRI never accepts tissue where clear consent for research has not been obtained. 4) Redact all incoming documents relating to donors. NDRI assigns an identification code to each donor to protect the donor’s identity. All samples remain coded and no information that could be used to identify the donor is ever released by NDRI. This process ensures that researchers receiving tissues cannot identify or contact the donor or donor’s family.

Our specific research project using the above tissues was approved by the Institutional Review Board of the University of Southern California who certified the study as exempt:45 CFR 46.101 (b) (4).

Finally, these same testis samples were also used in earlier studies published in PLOS Genetics [21]. No donors were accepted if they had been treated with drugs known to interfere with normal spermatogenesis. All samples were frozen within 10–12 h after death.

### Testis Dissection

The testis dissection scheme has previously been described in detail, see Fig 1 in [6]. The epididymis was removed from the testis. Each testis was fixed in 70% ethanol at 4°C for about 3 days, cut into 6 slices and each slice further divided into 32 pieces of approximately equal size. Each testis piece, and its subsequent DNA sample, contained both germline (90%) and somatic cells (10%) cells. See http://training.seer.cancer.gov/anatomy/reproductive/ for an introduction to human testis anatomy.

### DNA purification and quantitation

DNA was extracted from each piece using a Puregene kit (Gentra Systems). The concentrations of all DNA samples were determined by real-time PCR on a Bio-Rad Opticon 2 instrument [6]. On average, each testis piece contained DNA from about 2.5x10^7^ cells (range 1.5x10^7^ to 4.1x10^7^). This estimate was made using the average number of genomes per testis piece and data on the number of germline and non-germline cells found in individual human testes [27] after accounting for the ploidy of pre- and post-meiotic germline cells as well as non-germline cells (Sertoli cells). Purified human blood DNA was purchased from Promega.

### Sequencing library production

The 32 pieces from slice1 of testis #60891 were used to prepare Safe Sequencing System (SSS) libraries from a portion of the *FGFR3* gene. Slice 3 of this same testis was used to prepare SSS libraries from a portion of the *MECP2* gene. Libraries of a coding segment of the *PTPN11* gene from each of three pieces of a 36 year old donor’s testis (#62923, slice 5) were also prepared.

#### FGFR3

A million human genomes (~3 ug) from each testis piece were uniquely tagged immediately 5’ and 3’ of the *FGFR3* target coding region. The forward PCR primer (S1 Fig) carried a portion of the Illumina sequencing primer region, the universal identifiers (UIDs) and finally a sequence that targets the PCR to a 26 bp downstream *FGFR3* segment. The reverse primer (S1 Fig) contained a portion of the Illumina sequencing primer region, a barcode designating the testis piece number, a different UID and a different *FGFR3* sequence that targets the PCR to a 23 bp upstream *FGFR3* region. Between the 3’ ends of these two primers is a 171 bp *FGFR3* sequence that is the target of NGS. This primer pair was used for two cycles of PCR in Round 1. Each of the 11 reactions used (per piece) contained ~0.9x10^5^ genomes of purified DNA, 1x GC Phusion Buffer (1.5 mM MgCl_2_), additional MgCl_2_ for a final concentration of 3.5 mM, 300 nM of each of the first round primers (SM Fig 1), 80μM dNTP, Phusion Hot Start polymerase (0.02U/ul) and 0.26x SYBR green, 2.6 μM ROX solution in a 45ul reaction. Note that in the presence of SYBR green I, the type-specific background mutation frequencies were the same as that found when no SYBR green I was added (see S3 Table). After a 98°C 5 min initial denaturation two cycles were run (98°C 30sec, 71°C 4min, 72°C 1min) followed by 72°C for 5 minutes in a MJ Research Opticon 2 QPCR instrument. The PCR product in each reaction was separated from unused primers by adding 0.67x volume of Agencourt AMPure XP magnetic beads (Beckman Coulter) to remove the first round primers. After 5 minutes at room temperature, the beads were placed on the magnet for 2 minutes, washed twice with 70% ethanol, and allowed to dry for 6–7 minutes in a biosafety cabinet.

The DNA on the dried magnetic beads was resuspended in the second round PCR buffer which was identical to the first round buffer except for 550 nM of each second round primer (S1 Fig) also in a 45ul reaction. Twenty-eight additional cycles of PCR were performed (98°C 5 min initial denaturation, 98°C 10sec, 72°C 15sec ending with 72°C for 5 min). Both second round primers contained sequences complementary to the 5’ most portion of the first round PCR product at the 3’ end. The 5’ end of the second round primers carried the sequences needed for binding to the Illumina flowcell.

Following the second round, 0.67x PEG 8000 (20%) dissolved in 2.5M NaCl (required for the bead-DNA binding step) was added to each reaction, allowed to sit at room temperature for 5 minutes, exposed to the magnet for 2 minutes, washed twice with 70% ethanol, and allowed to dry for 6–7 minutes. The DNA was eluted in 10 ul of H_2_O and the expected 367 bp final products from each of the eleven reactions for each piece were pooled. For the final library, all 32 of the different pooled PCR products were combined in equal amounts based on qPCR. The pooled and concentrated (SpeedVac) sample was evaluated (Bioanalyzer 2100, Agilent Technologies) to quantify the final amount of DNA in the library. The DNA was then loaded on a HiSeq 2000 and 100 bp paired-end sequencing was performed.

#### MECP2

For each of the 32 testis pieces, six reactions, each containing 170,000 genomes, were subjected to two rounds of SSS PCR (one million genomes per piece total). For the 1^st^ PCR round, each 100ul amplification reaction contained 1X Phusion HF buffer, 1.5 mM MgCl2, 62.5 uM of dNTPs, 115nM of each forward and reverse strand primer (S1 Fig) 0.4X SYBR Green, 4 uM Rox, and 0.04 unit/ul of Phusion Hot Start DNA polymerase (Thermo Scientific). PCR was carried out in 96 well plates using a MJ Research Opticon 2. The cycling conditions were an initial denaturation for 6 min, 98°C and two cycles of 100°C for 15s, 69°C for 8min and 72°C for 1min followed by 10min at 72°C. In order to remove the first round primers, the single-stranded DNA in each well was digested with 60 units of Exonuclease I (Lucigen) at 37°C for 80 min followed by a 5 min heat inactivation at 98°C. For the second round an additional 30 cycles were performed by adding the same 2^nd^ round primers used for *FGFR3* to a final concentration of 500nM. The cycling conditions were thirty cycles of 98°C for 10s, 72°C for 15s. Following the 2^nd^ round, PCR product from each of the six reactions were captured using 0.68x beads and eluted in 30ul of H_2_O. Three ul from each of the six reactions were loaded into a different well of a 6% PAGE gel to confirm the purity of the final PCR product. Total DNA concentration for the piece was measured by nanodrop.

The SSS library was made by pooling the same amount of DNA from each of the 32 pieces, concentrating the mixture (1 hr SpeedVac) and quantitating the amount of DNA with an Agilent 2100 Bioanalyzer before loading on the Illumina instrument.

#### PTPN11

DNA from three pieces (testis #62923) were used. Each of the three pieces were divided into three aliquots to create 9 SSS sequencing libraries. The first aliquot from each piece was made into three distinct libraries. These three libraries (#1–3) were prepared along the lines described for *MECP2*. For each library a total of 250,000 genomes from one piece were divided into 20 wells (12,500 genomes each). The two cycles of the first round PCR reactions, (50ul total) contained 1X Phusion HF buffer (1.5 mM MgCl_2_), additional MgCl_2_ for a final concentration of 3.5 mM, 62.5 uM of each dNTP, 220 nM of each *PTPN11* primer (S1 Fig), 4 uM Rox, 0.4 X SYBR Green I, 0.04 unit/ul of Phusion Hot Start DNA polymerase. The cycling conditions were initial denaturation 1 min, 98°C followed by 100°C for 15s, 68°C for 7min and 72°C for 1min followed by 10min, 72°C. For the second round the initial Exo I treatment, PCR primers, reagents, cycling conditions and times were identical to those used for *MECP2*.

The remaining six libraries (#4–9), were also made using DNA from each of the original three pieces but by two slightly different protocols. The first alternative libraries (#4–6) also used a total of 250,000 genomes divided into 20 wells (12,500/well) but only the forward SSS primer (at 220 nM) was added for the first cycle. Otherwise the first cycle reagents, cycling conditions and incubation times were identical to those used for libraries #1–3. In addition, instead of continuing on to the second cycle the reaction was stopped and followed by Exo I digestion (and heat inactivation) before the second cycle was initiated with the addition of only the reverse primer (220 nM). The second cycle conditions were otherwise identical to the first cycle. Following the end of the second cycle was another Exo I digestion and inactivation step. This was followed by the second round of 26 PCR cycles with the same reagents and conditions and a similar magnetic bead purification (0.70x) used for libraries #1–3.

The second alternative libraries (#7–9) were identical to the first alternatives except only the reverse primer was used in the first cycle and only the forward primer was used in the second cycle. Each of the nine libraries were analyzed separately. Fifteen percent of each second round PCR product was loaded on a 6% PAGE gel and the correct size confirmed. Each PCR product was measured by nanodrop and the nine libraries were pooled using the same amount of DNA from each of the 9 final 2^nd^ round products. 90 ul of this pooled sample was loaded on to a 3% agarose gel run at 90V for 1hr and stained with 0.01% SafeView (Applied Biological Materials Inc). The fragment of the correct size was cut out of the gel and DNA recovered using a Zymo clean gel DNA recovery kit (ZYMO RESEARCH). The final amount of DNA in the library mixture was measured with an Agilent 2100 Bioanalyzer.

### Barcodes

We selected the barcode sequences for the different pieces such that if there is a mistake at any one site in the barcode then we can infer the correct barcode, and if there is a mistake at two base pairs then we will know there has been a mistake and it will not be incorrectly counted as a different barcode (e.g., [28]).

### Quantitative methods

We wrote Perl scripts to analyze the raw sequencing data. We only considered reads with fewer than 5% of bases different from the *FGFR3*, *MECP2* or *PTPN11* target sequences. To measure the sequencing error, we compared base pairs where reads #1 and #2 overlapped. If these two reads differed and one of the reads agreed with the reference, we assumed the other read was a sequencing mistake (the number of cases where both reads disagreed with the reference was too few to affect the sequencing error frequencies). To form UIDs, we only considered those reads where all the base pairs in the barcode and UID sequence had quality scores of 32 or greater. We clustered reads with the same barcode and UID into UID families. We only considered families with at least 3 paired reads with the same UID. We only considered the base pairs in these families with at least 3 reads with quality scores 32 or greater (reads with quality scores less than 32 at a base pair were not considered for that particular base pair). A super-mutant is when 95% or more of the reads in a UID family agree with each other and disagree with the reference at a site. For the Separate method, we analyzed the two strands separately. S7 Fig shows the distribution of UID family sizes. S8 Fig shows the distribution of pairwise sequence differences between UID sequences.

To measure PCR errors, we only considered base pairs where reads #1 and #2 overlapped and agreed. We considered UID families of size 2 to 10 (previously we considered families of size at least 3). We measured the frequency of mutations present in only one read (these are not super-mutants). The Perl scripts we wrote outputted tables of numbers that were then read and statistically analyzed in R.

## Supporting Information

S1 Fig All SSS primer sequences. (PDF)

S2 Fig *MECP2* mutation frequencies color-coded by reference base. Each dot represents the average of 32 different testis pieces. Red indicates an A or T base, blue indicates a C or G (non-CpG) base, and green indicates a CpG. The mutation frequency is the sum of all mutations at each site, so, e.g., if a site is a C, the mutation frequency is the sum of the C>A, C>G, and C>T frequencies at that base. The 95% confidence interval for each position is also shown.
(PDF)

S3 Fig *FGFR3* mutation frequencies color-coded by reference base. There are two separate plots because unlike the *PTPN11* and *MECP2* experiments, there is an un-sequenced gap between the paired reads. Each dot represents the average of 32 different testis pieces. Red indicates an A or T base, blue indicates a C or G (non-CpG) base, and green indicates a CpG. The mutation frequency is the sum of all mutations at each site, so, e.g., if a site is a C, the mutation frequency is the sum of the C>A, C>G, and C>T frequencies at that base. The 95% confidence interval for each position is also shown.
(PDF)

S4 Fig Block drawing of Separate method. Shown is a general outline of the steps for each pair of Separate reactions.
(PDF)

S5 Fig Separate method and DNA damage during the first PCR cycle. The Separate method separately amplifies the coding and non-coding strands. The aliquot on the left-hand side only amplifies the top strand, while the aliquot on the right-hand side only amplifies the bottom strand. The yellow rectangles indicate strands that are not primer extension templates and are removed by exonucleases. The red lightning bolts represent DNA deamination (or oxidation) during the first PCR cycle. The Separate method detects a C>T mutation on the left-hand side, and no mutation on the right-hand side. As shown in S6 Fig this is balanced by DNA damage during the second PCR cycle.
(PDF)

S6 Fig Separate method and DNA damage during the second PCR cycle. The Separate method separately amplifies the coding and non-coding strands. The aliquot on the left-hand side only amplifies the top strand, while the aliquot on the right-hand side only amplifies the bottom strand. The yellow rectangles indicate strands that are not primer extension templates and are removed by exonucleases. The red lightning bolts represent DNA deamination (or oxidation) during the second PCR cycle. The Separate method detects a G>A mutation on the right-hand side, and no mutation on the left-hand side. As shown in S5 Fig this is balanced by DNA damage during the first PCR cycle.
(PDF)

S7 Fig Log-log histogram of UID family sizes. Note the larger family sizes in the *PTPN11* experiment compared to the *MECP2* and *FGFR3* experiments.
(PDF)

S8 Fig Distribution of pairwise sequence differences between UID sequences. The *PTPN11* experiment has a UID with 21 bases while the *MECP2* and *FGFR3* experiments have UIDs with 20 bases. Note the similarity of the distributions to the theoretical distributions for random UIDs of the same length.
(PDF)

S1 Table Comparison of somatic (blood) to germline mutation frequencies. (PDF)

S2 Table Mutation frequency as a function of mutation type and loci. (PDF)

S3 Table No significant difference (p-value 0.65) when comparing the mutation frequency in experiments with and without SYBR green. (PDF)

S1 Text Mathematical calculations for deep sequencing experimental design. (PDF)
