# Supplementary material for: Estimating Exceptionally Rare Germline and Somatic Mutation Frequencies via Next Generation Sequencing
Source: PLoS One. 2016 Jun 24;11(6):e0158340. doi: 10.1371/journal.pone.0158340 (PMC4920415; doi:10.1371/journal.pone.0158340)
Supplement: S1 Fig — (PDF) [file pone.0158340.s001.pdf]

S1 Fig. First round forward and reverse primers for the *FGFR3*, *MECP2*, and *PTPN11* experiments, and second round forward and reverse universal primers.

A. First round forward *FGFR3* primer (Illumina sequencing primer in red)

5' CGACGCTCTTCCGATCTNNNNNNNNNNNNNNGACGTGCACAACCTCGACTACTACAA 3'

B. First round reverse *FGFR3* Primer (example of barcode)

5' CTCGGCATTCCTGCTGAACCGCTCTTCCGATCTCAGACTATNNNNNNGAAGCCTGAG CTCTGCAGGACAC 3'

C. Second round forward primer (*=phosphorothioate bond, Illumina flowcell sequence in blue)

5' AATGATACGGCGACCACCGAGATCTACACTCTTTCCCTACACGACGCTCTTCCGAT*C*T 3'

D. Second round reverse primer

5' CAAGCAGAAGACGGCATACGAGATCGGTCTCGGCATTCCTGCTGAACCGCTCTTCCG*A*T 3'

E. First round forward *MECP2* Primer

5' CGACGCTCTTCCGATCTNNNNNNNNNNNNNNGACACATCCCTGGACCCTAATGATTTTG 3'

F. First Round Reverse *MECP2* Primer (example of barcode)

5' CTGAACCGCTCTTCCGATCTTGTGTATCNNNNNNNGGGAGATTTGGGCTTCTTAGGTGGTTT 3'

G. First Round Forward *PTPN11* Primer

5' CGACGCTCTTCCGATCTNNNNNNNNNNNNNNNGTCCAGGACTTATGTGACCGTGGTCT 3'

H. First Round Reverse *PTPN11* Primer (example of barcode)

5' AACCGCTCTTCCGATCTAGTAGGNNNNNNNAACACTGTGAAAAGCAAAGCTTAC 3'
