# Supplementary figures and images for: Estimating Exceptionally Rare Germline and Somatic Mutation Frequencies via Next Generation Sequencing
Source: PLoS One. 2016 Jun 24;11(6):e0158340. doi: 10.1371/journal.pone.0158340 (PMC4920415; doi:10.1371/journal.pone.0158340)

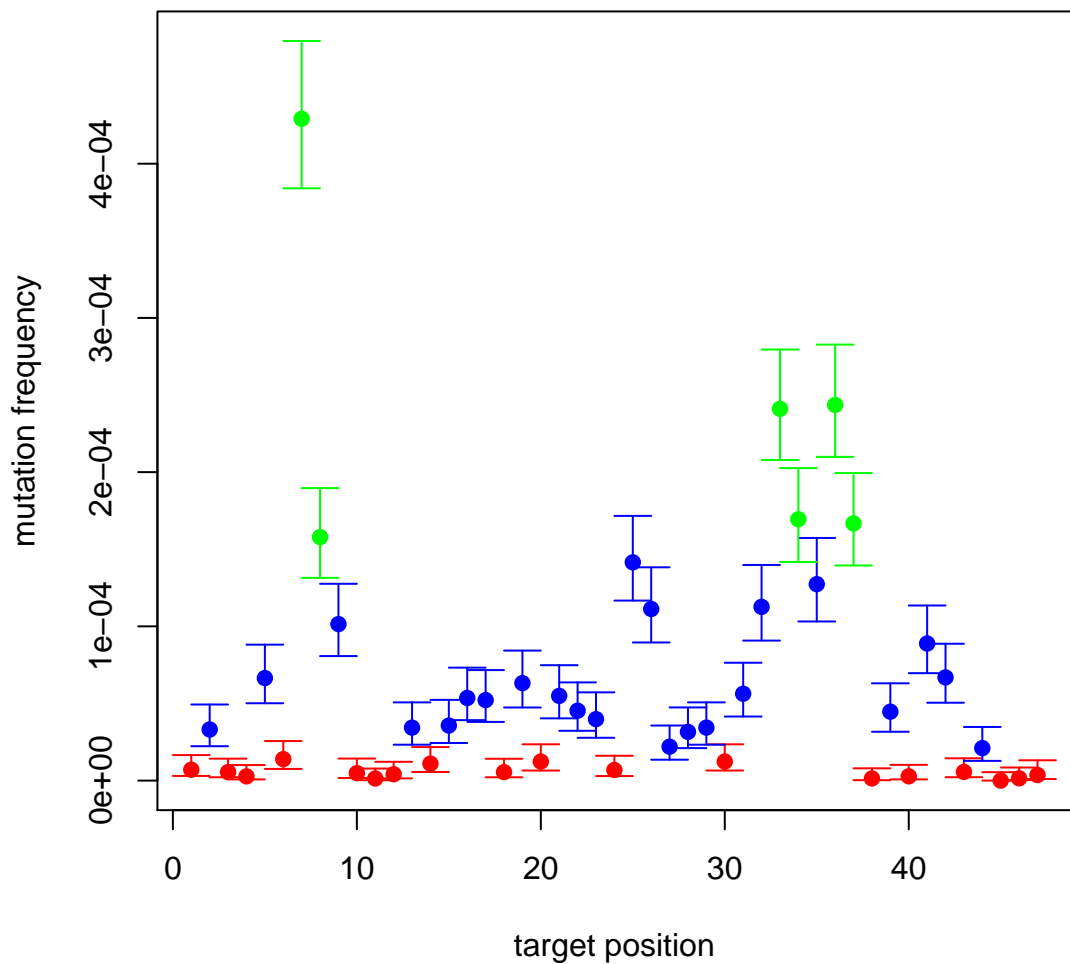

Supplement: S2 Fig — Each dot represents the average of 32 different testis pieces. Red indicates an A or T base, blue indicates a C or G (non-CpG) base, and green indicates a CpG. The mutation frequency is the sum of all mutations at each site, so, e.g., if a site is a C, the mutation frequency is the sum of the C>A, C>G, and C>T frequencies at that base. The 95% confidence interval for each position is also shown. (PDF) [file pone.0158340.s002.pdf]

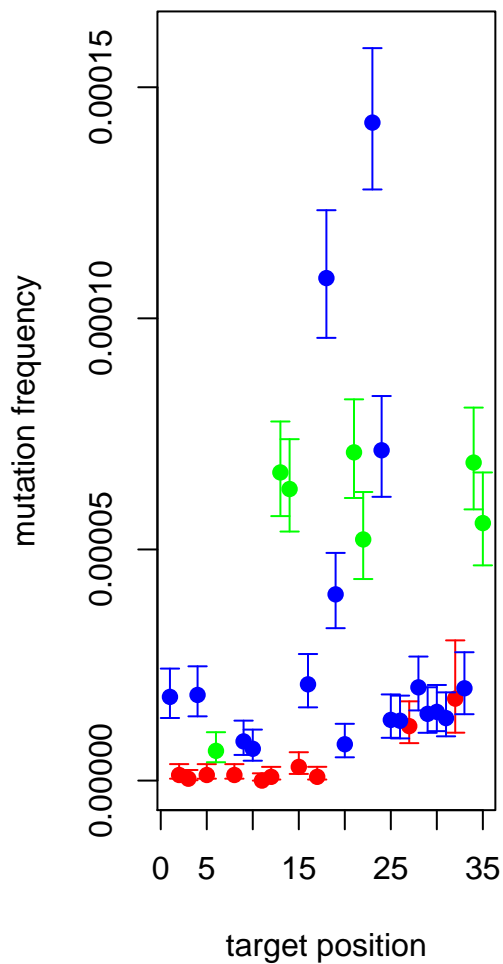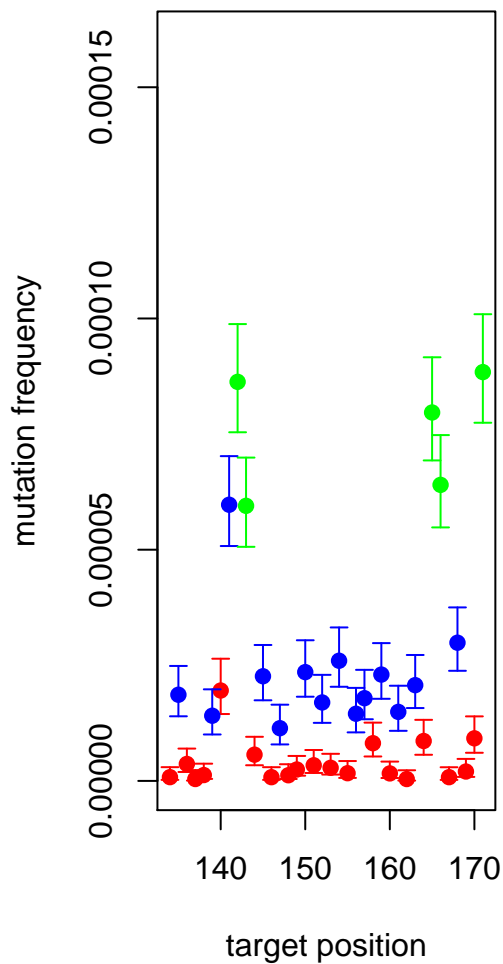

Supplement: S3 Fig — There are two separate plots because unlike the PTPN11 and MECP2 experiments, there is an un-sequenced gap between the paired reads. Each dot represents the average of 32 different testis pieces. Red indicates an A or T base, blue indicates a C or G (non-CpG) base, and green indicates a CpG. The mutation frequency is the sum of all mutations at each site, so, e.g., if a site is a C, the mutation frequency is the sum of the C>A, C>G, and C>T frequencies at that base. The 95% confidence interval for each position is also shown. (PDF) [file pone.0158340.s003.pdf]

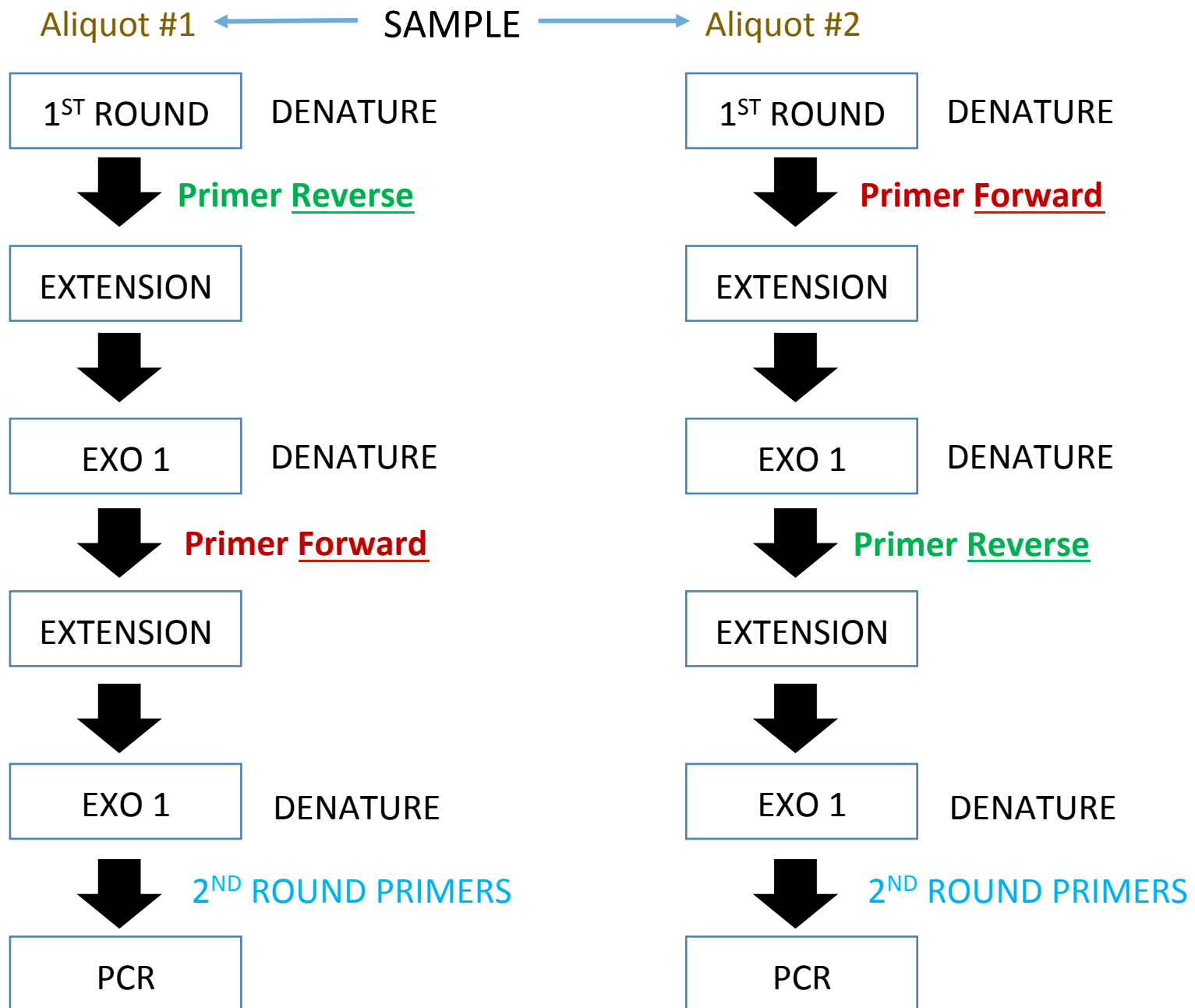

Supplement: S4 Fig — Shown is a general outline of the steps for each pair of Separate reactions. (PDF) [file pone.0158340.s004.pdf]

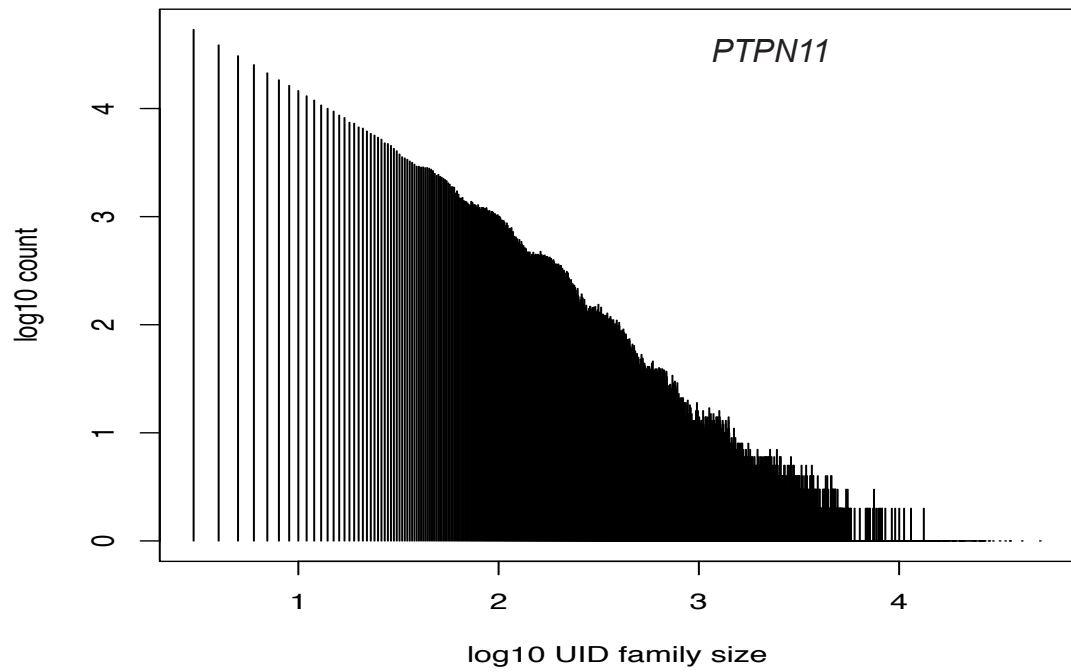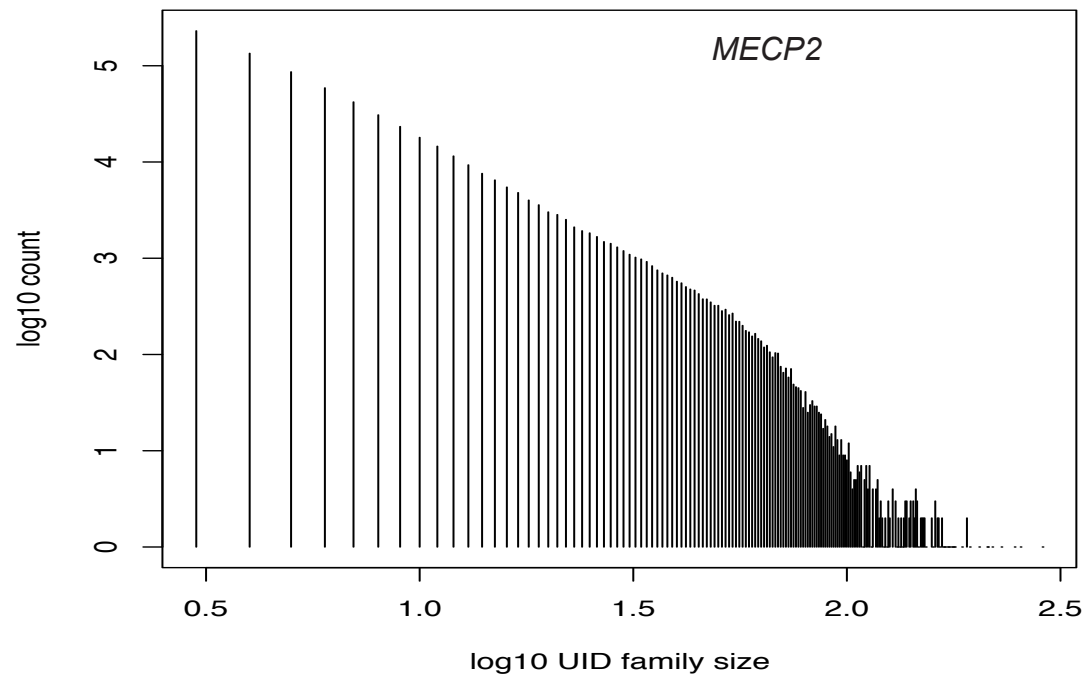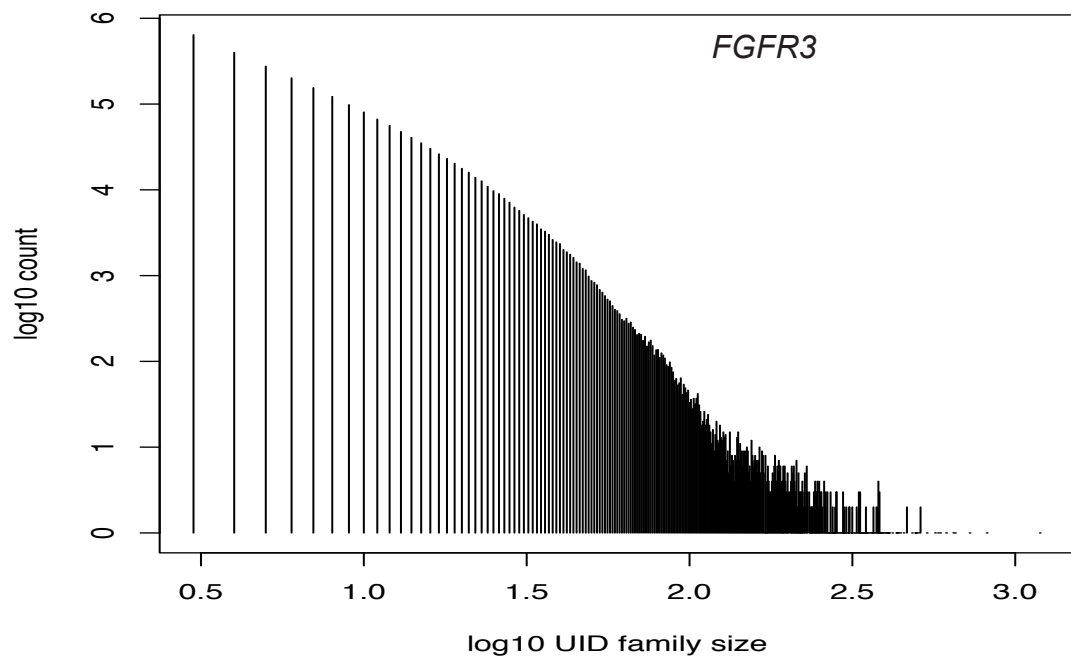

Supplement: S7 Fig — Note the larger family sizes in the PTPN11 experiment compared to the MECP2 and FGFR3 experiments. (PDF) [file pone.0158340.s007.pdf]

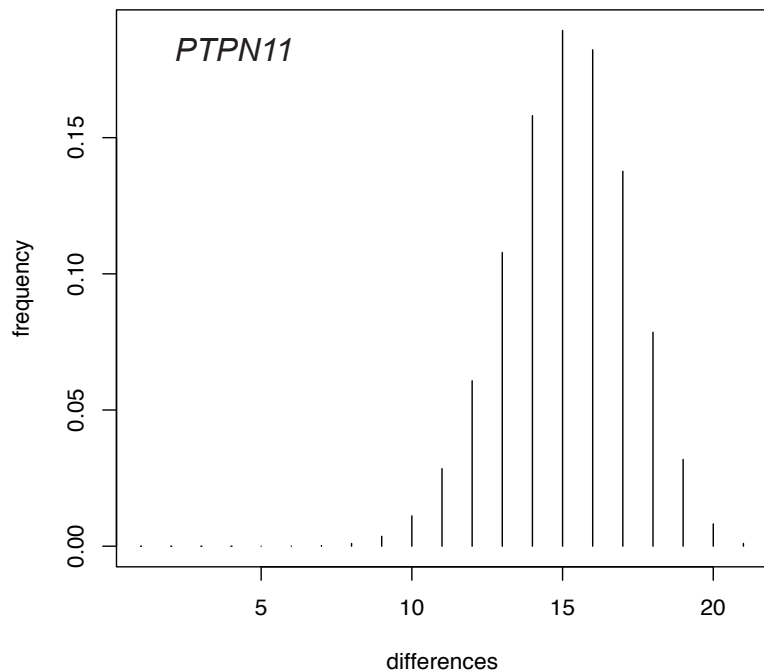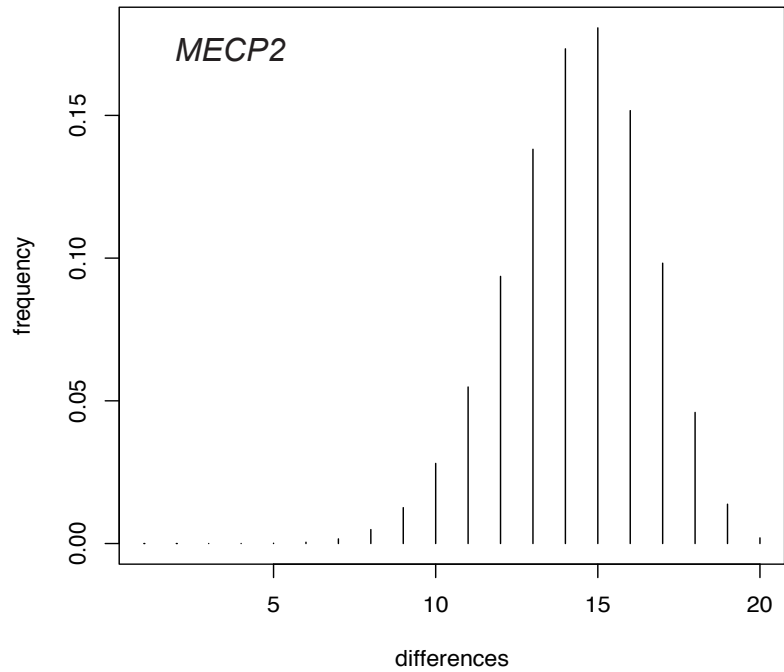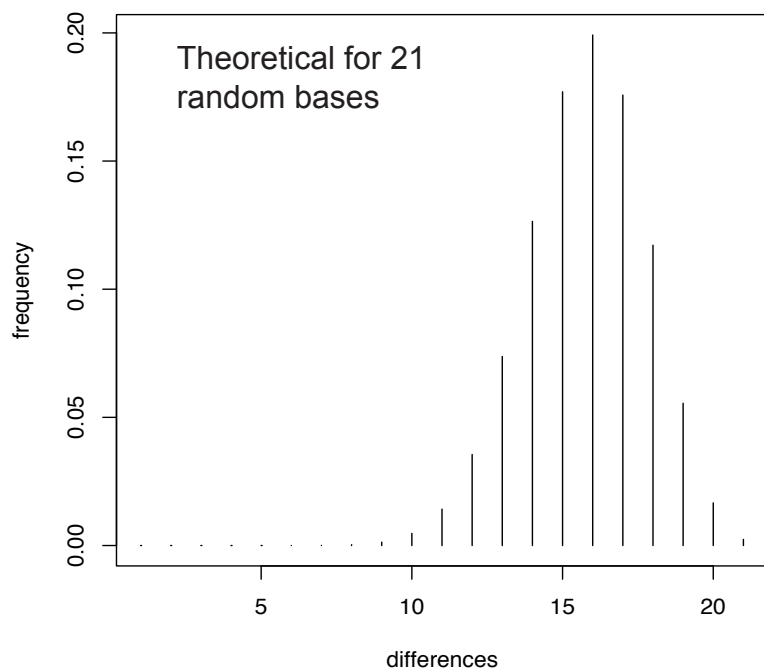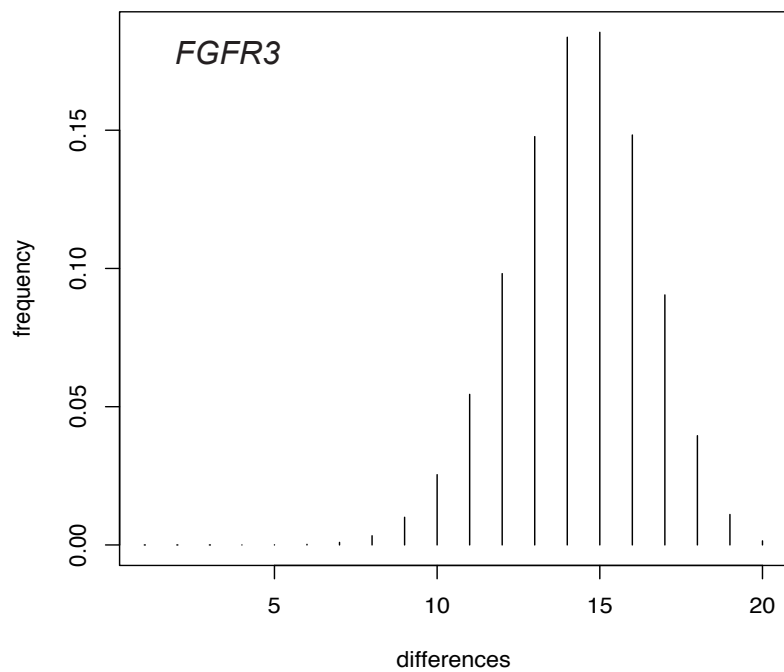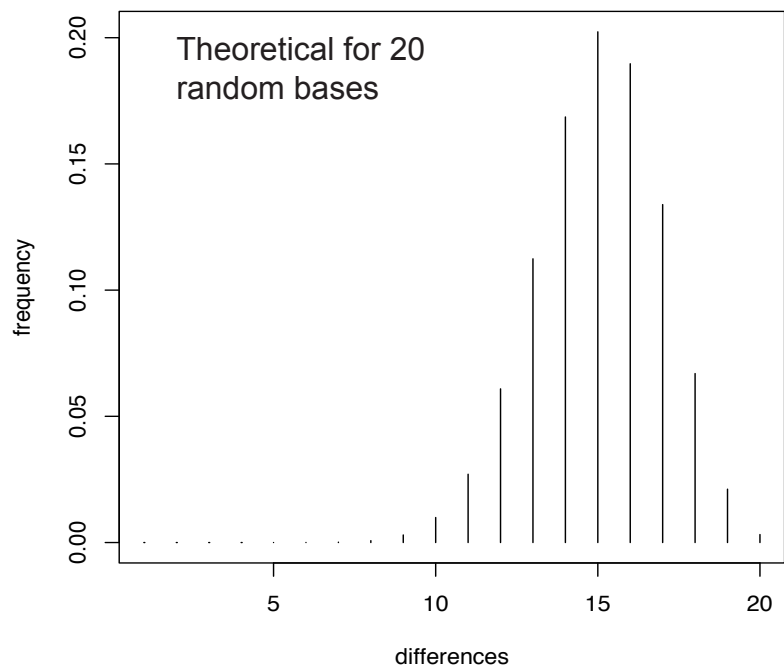

Supplement: S8 Fig — The PTPN11 experiment has a UID with 21 bases while the MECP2 and FGFR3 experiments have UIDs with 20 bases. Note the similarity of the distributions to the theoretical distributions for random UIDs of the same length. (PDF) [file pone.0158340.s008.pdf]
