# Supplementary material for: Estimating Exceptionally Rare Germline and Somatic Mutation Frequencies via Next Generation Sequencing
Source: PLoS One. 2016 Jun 24;11(6):e0158340. doi: 10.1371/journal.pone.0158340 (PMC4920415; doi:10.1371/journal.pone.0158340)
Supplement: S5 Fig — The Separate method separately amplifies the coding and non-coding strands. The aliquot on the left-hand side only amplifies the top strand, while the aliquot on the right-hand side only amplifies the bottom strand. The yellow rectangles indicate strands that are not primer extension templates and are removed by exonucleases. The red lightning bolts represent DNA deamination (or oxidation) during the first PCR cycle. The Separate method detects a C>T mutation on the left-hand side, and no mutation on the right-hand side. As shown in S6 Fig this is balanced by DNA damage during the second PCR cycle. (PDF) [file pone.0158340.s005.pdf]

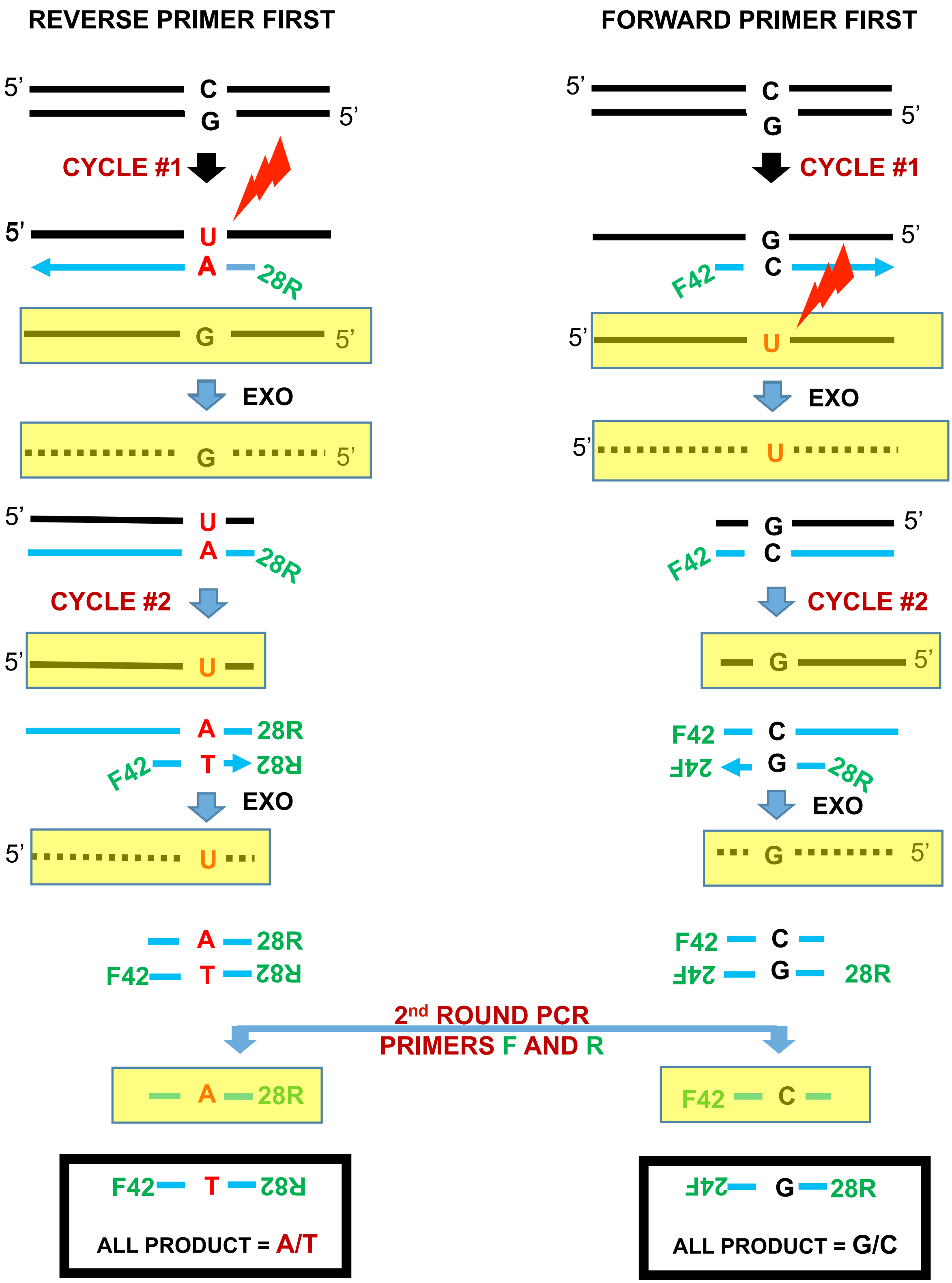
REVERSE PRIMER FIRST
FORWARD PRIMER FIRST
CYCLE #1
EXO
CYCLE #2
28R
F42
2nd ROUND PCR
PRIMERS F AND R
ALL PRODUCT = A/T
ALL PRODUCT = G/C
