# Supplementary material for: Estimating Exceptionally Rare Germline and Somatic Mutation Frequencies via Next Generation Sequencing
Source: PLoS One. 2016 Jun 24;11(6):e0158340. doi: 10.1371/journal.pone.0158340 (PMC4920415; doi:10.1371/journal.pone.0158340)
Supplement: S1 Table — (PDF) [file pone.0158340.s009.pdf]

Table S1. Comparison of somatic (blood) to germline mutation frequencies

	A or T mutation frequency	C or G mutation frequency
Somatic (blood)	$4.5x10^{-6}$	$2.8x10^{-5}$
Germline	$8.7x10^{-6}$	$8.0x10^{-5}$

Data from *PTPN11* experiments
