# Supplementary material for: Estimating Exceptionally Rare Germline and Somatic Mutation Frequencies via Next Generation Sequencing
Source: PLoS One. 2016 Jun 24;11(6):e0158340. doi: 10.1371/journal.pone.0158340 (PMC4920415; doi:10.1371/journal.pone.0158340)
Supplement: S2 Table — (PDF) [file pone.0158340.s010.pdf]

Table S2. Mutation frequency as a function of mutation type and loci

Mutation type	Mutation frequency *PTPN11* experiment	Mutation frequency *MECP2* experiment	Mutation frequency *FGFR3* experiment
A>C/T>G	$6.4x10^{-7}$	$3.0x10^{-7}$	$3.8x10^{-7}$
C>G/G>C	$2.6x10^{-6}$	$1.1x10^{-6}$	$7.5x10^{-7}$
A>T/T>A	$2.9x10^{-6}$	$1.0x10^{-6}$	$4.7x10^{-7}$
A>G/T>C	$4.6x10^{-6}$	$4.2x10^{-6}$	$2.6x10^{-6}$
C>T/G>A (non-CpG)	$3.6x10^{-5}$	$2.7x10^{-5}$	$1.0x10^{-5}$
G>T/C>A	$3.1x10^{-5}$	$4.2x10^{-5}$	$1.6x10^{-5}$
C>T/G>A (CpG)	$2.2x10^{-4}$	$1.9x10^{-4}$	$5.0x10^{-5}$
