# Supplementary material for: Estimating Exceptionally Rare Germline and Somatic Mutation Frequencies via Next Generation Sequencing
Source: PLoS One. 2016 Jun 24;11(6):e0158340. doi: 10.1371/journal.pone.0158340 (PMC4920415; doi:10.1371/journal.pone.0158340)
Supplement: S3 Table — (PDF) [file pone.0158340.s011.pdf]

Table S3. No significant difference (p-value 0.65) when comparing the mutation frequency in experiments with and without SYBR green

	Mutation frequency	95% confidence interval
Without SYBR green	$2.2x10^{-6}$	$1.2\text{-}3.5x10^{-6}$
With SYBR green	$2.6x10^{-6}$	$1.2\text{-}4.4x10^{-6}$

Data from *MECP2* experiments
