# Supplementary material for: Estimating Exceptionally Rare Germline and Somatic Mutation Frequencies via Next Generation Sequencing
Source: PLoS One. 2016 Jun 24;11(6):e0158340. doi: 10.1371/journal.pone.0158340 (PMC4920415; doi:10.1371/journal.pone.0158340)
Supplement: S1 Text — (PDF) [file pone.0158340.s012.pdf]

S1 text. Mathematical calculations for experimental design.

We use the symbol (a,b) for the mathematical combination symbol (e.g., [1]) read "a choose b." Many of the calculations below use the Poisson approximation (e.g., [1]). For the different experiments the conditions differ, we consider representative or conservative values. Let m be the number of distinct molecules that have UIDs and barcodes attached in the first round of PCR (each original molecule may yield 0, 1, or 2 such molecules after the first two PCR cycles). Let n be the length of the UID sequence, then 4^n is the number of UID sequences. Let r be the number of quality aligned reads, u be the number of UID families, f be a UID family size, s be the sequencing error rate per site, and c be the number of PCR cycles in the second round of PCR.

1. How often are two distinct molecules are assigned the same UID sequence?

The number of pairs of distinct molecules assigned the same UID is Poisson with mean (m,2) / 4^n. Consider distinct molecules m = 1 million per testis piece (different pieces have different barcodes) and UID length n = 20. Then the mean number of such pairs is 0.5.

2. How often do we expect two distinct UID families to be assigned UID sequences that differ at exactly one nucleotide position? If there is a mistake in a UID sequence (either during sequencing or PCR amplification) how likely is this mistaken UID to be the same as a UID assigned to another DNA molecule?

The number of pairs of UID families assigned UID sequences that differ by exactly one base in their UID sequence is Poisson with mean (u,2) [n $(0.25)^{n-1}$ 0.75]. Consider UID families u = 100

thousand and UID length n = 20. Then the mean number of such pairs is 0.3. For a mistake to have this effect it would have to happen in one of such a pair of UID families and at the particular site in the UID sequence where they differ.

3. What is the probability one particular DNA molecule is incorrectly represented by multiple UID families?

During the first round of PCR when the UIDs and barcodes are being attached, each molecule may yield up to 2 molecules with distinct UIDs. As was shown in #2, a mistake in the UID is very likely to produce a new UID sequence. The same sequencing mistake in the UID sequence for multiple members of a UID family can create another UID family. The probability that 3 members have the same sequencing mistake is $(f,3)\ s^3\ (1/3)^2\ n$. Consider family size f = 100, sequencing error rate $s = 5.3\text{x}10^{-6}$, and UID length n = 20. Then this probability is $5\text{x}10^{-11}$. The probability that 4 members have the same mistake is even smaller. Now consider a much larger family size f = 15,000 with the same sequencing error rate and UID length. The probability 3 members have the same sequencing mistake increases to $1.9\text{x}10^{-4}$. If in addition to increasing the family size we also increase the sequencing error rate $s = 5.9\text{x}10^{-5}$ (allowing reads with lower quality scores in the UID sequence) the probability 3 members have the same sequencing mistake now increases to 0.26.

A mistake in the UID sequence during a PCR cycle in the second round can also create another UID family. If this mistake is early in the second round then it is more likely that there will be enough sequenced reads to create two UID families then if this mistake is late in the second round. It is difficult to calculate the probability of this problem happening. One strategy that we have tried is if there are multiple UID families that differ at only one base in the UID

sequence, then we only keep the largest such UID family and remove the rest. This removes a relatively small number of UID families and does not affect the average mutation type frequencies, but does affect the mutation frequencies at a small number of testis piece and nucleotide position combinations (especially if there are very large UID families).

4. How likely is it that two distinct UID families are assigned the same long or short partial UID sequence?

Let n1 represent the length of the partial UID sequence. Then the number of pairs of UID families with the same partial UID sequence is Poisson with mean (u,2) / ($4^{\wedge n1}$). Consider UID families u = 100,000. For the short UID length n1 = 7, the mean number of such pairs is 305,173. For the long UID length n1 = 14, the mean number is 19.

5. What is the probability a mistake in the target genomic sequence during one of the early cycles of the second round of PCR creates an erroneous super-mutant?

A PCR mistake in the target genomic sequence during one of the early cycles of the second round of PCR that is in a common branch shared by all members of a UID family would erroneously create a super-mutant. Let's assume every molecule amplifies in every PCR cycle. Consider two members of a UID family. With probability 0.5 they have no common branches in the second round of PCR, with probability 0.5^2 they have one common branch at the beginning of the second round, with probability 0.5^3 they have 2 common branches at the beginning of the second round, etc. Then for c = 30 PCR cycles the mean number of common branches is 1. For three or more reads the mean number of common branches is less.

6. How can two mistakes during PCR erroneously create a super-mutant?

Imagine two PCR mistakes occur during the second round of PCR. The first mistake occurs in the target genomic sequence. The second mistake occurs in one of the molecules with the first mistake. This second mistake is in the UID sequence. If enough reads have both mistakes to form a UID family, then the first mistake will appear as a super-mutant in this new family. Due to the discussion in #5, there are few common branches for such mistakes to have this effect. As in #3, we removed UID families whose UID sequences differed by only base from other UID families.

A similar scenario that can erroneously create a super-mutant is again if there are two mistakes during the second round. Imagine first there is a PCR mistake in the target genomic sequence. Later in one of the molecules with the first mistake there is a PCR jumping event. PCR jumping is when primer extension is incomplete in one cycle and the incomplete PCR product then acts as a primer in a subsequent cycle. Since our UID is split between the two ends of the paired read, such a jumping event will combine the partial UID on one side from one family with the partial UID on the other side from a different family to create a new UID sequence. The first mistake will appear as a super-mutant in this new family. Unfortunately, we do not have a good way to estimate PCR jumping rates with this data. One way to do this would be if the barcodes were split between the two ends. As a proxy, if there were multiple families with the same long partial UID but paired with different short UIDs we tried keeping only the largest such UID family. This strategy had minimal effect on mutation frequencies.

7. How many UID families can we expect as a function of the numbers of initial molecules and sequenced reads?

It depends on the threshold for the number of reads to count as a UID family, in this manuscript we used a threshold of 3. Let's assume that each of the distinct molecules that have barcodes and UIDs attached in the first round of PCR are equally amplified in the second round of PCR. For any particular such molecule the size of its UID family is Poisson with mean $v = r / m$. The probability this family is size 3 or greater is $p = 1 - \exp(-v) - [v \exp(-v)] - [v^2 \exp(-v) / 2]$. Then the expected number of UID families of size 3 or more is m p. Consider distinct molecules m = 32 million and sequenced reads r = 28 million, the expected number of UID families is 1.9 million. The previous numbers correspond to the *FGFR3* experiment, where the observed number of UID families is 2.5 million.

Unlike the *FGFR3* experiment, for the *MECP2* and *PTPN11* experiments, when using the number of initial molecules from Table 4 for m the predicted number of UID families is quite different than what we observe. If instead we use one-quarter of the number of initial molecules from Table 4 for m, due to not all initial molecules having a UID and barcode attached during the first round of PCR, then the predicted and observed numbers of UID families are close.

8. What is the optimal utility ratio? What is the relationship between the number of initial molecules and the number of reads to achieve this ratio?

As in #7 it depends on the number of reads required to form a UID family, in this manuscript we required 3 reads. The formula in #7 shows that the optimal utility ratio (r / u) is 5.15. Moreover, this optimum is achieved when the ratio r / m is 3.3.

We would like to emphasize that r is the number of quality reads, which may be less than the total number of reads (up to as much as a factor of 2 less in Table 4). And m is the number of distinct molecules that have UIDs and barcodes attached in the first round of PCR, which may be less than the number of initial molecules (up to a factor of 4 less based on the discussion in #7).

SUPPLEMENTAL REFERENCE

1. Ross S. A first course in probability. 4th ed ed. New York: Macmillan College Publishing Company; 1994.
